# Selective inhibition of aldosterone production modulates human adrenocortical (HAC15) cell fate

**DOI:** 10.3389/fendo.2026.1872432

**Published:** 2026-10-12

**Authors:** Kha Chin Long, Amnani Aminuddin, Najiah Ajlaa Ayub, Geok Chin Tan, A. Rahman A. Jamal, Nor Azian Abdul Murad, Nor Adzimah Johdi, Celso E. Gomez-Sanchez, Norlela Sukor, Morris J. Brown, Elena A. Azizan

**Affiliations:** 1 Department of Medicine, Faculty of Medicine, The National University of Malaysia (UKM), Kuala Lumpur, Malaysia; 2 Research Center, Hospital Tunku Ampuan Besar Tuanku Aishah Rohani, Universiti Kebangsaan Malaysia Specialist Children’s Hospital, Kuala Lumpur, Malaysia; 3 Department of Pathology, Faculty of Medicine, The National University of Malaysia (UKM), Kuala Lumpur, Malaysia; 4 UKM Medical Molecular Biology Institute (UMBI), The National University of Malaysia (UKM), Kuala Lumpur, Malaysia; 5 Gordon V. (Sonny) Montgomery Veterans Affairs Medical Center and Department of Pharmacology and Toxicology, University of Mississippi Medical Center, Jackson, MS, United States; 6 William Harvey Research Institute, Queen Mary University of London, London, United Kingdom

**Keywords:** adrenal cell, adrenal cell proliferation, aldosterone, aldosterone synthase, apoptosis, cell fate, zona glomerulosa

## Abstract

**Background:**

Aldosterone, produced in the zona glomerulosa (ZG) by aldosterone synthase (CYP11B2), regulates sodium balance and blood pressure. In adult adrenals, CYP11B2-expressing ZG cells are sparse, likely reflecting chronic suppression of the renin–angiotensin system under high-salt conditions. Whether reduced aldosterone synthesis directly influences ZG cell behavior remains unclear. This study examined the impact of CYP11B2 inhibition on human adrenal cell fate and function.

**Methods:**

CYP11B2 was inhibited in HAC15 cells using siRNA (siCYP11B2) or baxdrostat, a selective CYP11B2 inhibitor. Forty-eight hours post-treatment, steroid hormone levels, RNA sequencing profiles, and functional outputs (EZMTT metabolic assay, xCELLigence real-time cell analysis, and Annexin V/SYTOX flow cytometry for apoptosis/necrosis) were evaluated.

**Results:**

siCYP11B2 reduced aldosterone production by 70% ± 2.9% (*n* = 18, *p* < 0.001) and suppressed mRNA expression of *CYP11B2* (88% ± 10.8%), *HNRNPA1* (33% ± 17.6%), and *UHMK1* (42% ± 13.7%) (*n* = 9, *p* < 0.05). Transcriptomic analysis revealed differential expression signatures enriched in autophagy- and mitophagy-related pathways. Treatment with U0126 (MEK1/2 inhibitor) dose-dependently reduced angiotensin II-stimulated aldosterone and cortisol production (*n* = 12, *p* < 0.001), and SB203580 (p38 MAPK inhibitor) increased the aldosterone-to-cortisol ratio (4.5 ± 0.5-fold, *n* = 12, *p* < 0.001), at concentrations previously reported to inhibit (25–50 µM U0126) or induce autophagy (1 µM SB203580), respectively. EZMTT assays demonstrated increased metabolic activity in siCYP11B2-treated cells at 96 h (Δ0.141 ± 0.02 absorbance units, *p* < 0.001), which was not observed following baxdrostat treatment. Baxdrostat treatment altered overall cell-death responses (FC = 1.42 ± 0.05, *n* = 144 and *p* = 1.3 × 10^−10^); however, the relative distribution of apoptotic and necrotic populations varied across independent experiments.

**Conclusion:**

Reduced aldosterone synthesis is associated with transcriptional signatures involving autophagy- and mitophagy-related pathways. Pharmacological treatment using U0126 and SB203580 altered steroidogenic output independently of *CYP11B2* transcript expression, providing hypothesis-generating evidence linking cellular homeostasis and mitochondrial quality control processes to adrenal steroidogenesis. Furthermore, pharmacological CYP11B2 inhibition using baxdrostat produced variable alterations in cell-death profile, highlighting important considerations for long-term therapeutic targeting.

## 1 Introduction

Primary aldosteronism (PA) is a common curable form of secondary hypertension, with a conservative estimated prevalence of 4%–10% of patients with hypertension (1, 2). PA is characterized by autonomous aldosterone hypersecretion, leading to increased renal and intestinal sodium reabsorption and potassium secretion, and consequent hypertension, often accompanied by hypokalemia in more severe cases.

Under physiological conditions, aldosterone is synthesized in the zona glomerulosa (ZG), the outermost layer of the adrenal cortex, where the enzyme aldosterone synthase (CYP11B2) catalyzes the final steps of aldosterone biosynthesis. Aldosterone production is tightly regulated by angiotensin II (AngII) and extracellular potassium, which together reflect physiological demands for sodium homeostasis and intravascular volume control (3, 4). Beyond its endocrine role, the ZG is increasingly recognized as a dynamic compartment of the adrenal cortex, characterized by continuous cellular turnover, centripetal migration, and functional plasticity across cortical zones (5, 6).

In this context, the concept of ZG “integrity” warrants careful definition. It may refer to anatomical localization beneath the adrenal capsule, the presence of CYP11B2-expressing cells, or the capacity for aldosterone production. Emerging *in vivo* evidence indicates that reduced aldosterone synthesis does not necessarily result in the loss of a discrete ZG layer, but rather in adaptive remodeling of adrenal cell populations. For example, pharmacological inhibition of CYP11B2 with baxdrostat in primates has been associated with expansion of the subcapsular region in a renin-dependent manner, accompanied by only modest increases in apoptosis, consistent with compensatory zonal remodeling rather than structural ablation (7). Similarly, in Cyp11b2⁻/⁻ mouse models, a subcapsular cell layer persists but adopts an altered molecular identity, while apoptotic activity is redistributed toward deeper cortical regions, again supporting dynamic reorganization rather than the disappearance of the ZG (8, 9).

Human studies further suggest that chronic suppression of the renin–angiotensin–aldosterone system such as under conditions of sustained high sodium intake is associated with reduced *CYP11B2* expression and increased apoptotic signaling within the ZG (10). These observations highlight a complex interplay between steroidogenic activity, hormonal regulation, and adrenal zonation. However, the mechanisms linking aldosterone biosynthesis to adrenal cell fate decisions and tissue organization remain incompletely defined. This gap is particularly relevant in pathological contexts such as aldosterone-producing adenomas, where dysregulated steroidogenesis coexists with altered cellular behavior. We have previously identified ZG-enriched genes, including *DACH1* and *LGR5*, that are associated with reduced aldosterone production and modulation of cell fate pathways, including apoptosis and differentiation, potentially through TGF-β and WNT signaling networks (10, 11). These findings raise the possibility that perturbations in steroidogenic capacity may directly influence adrenal cell survival and phenotypic plasticity.

In the present study, we investigated whether reducing aldosterone synthesis alters human adrenal cell fate. Using complementary genetic (siRNA-mediated silencing of *CYP11B2*) and pharmacological (baxdrostat-mediated inhibition) approaches in HAC15 human adrenocortical cells, we examined the impact of impaired steroidogenesis on cellular outcomes, including viability and programmed cell death. By integrating these approaches, our study aimed to examine how suppression of aldosterone synthesis is associated with changes in adrenal cellular behavior and to identify candidate molecular pathways that may link steroidogenic activity with cellular homeostasis.

## 2 Materials and methods

### 2.1 Cell culture maintenance and media

The HAC15 human adrenocortical carcinoma cell line (gifted by Professor Dr. William Rainey, Georgia Regents University, USA), a sub-clone of H295R, was confirmed to have the *CTNNB1* S45P mutation reported in the parent cell line (12) and was cultured in Dulbecco’s Modified Eagle’s Medium/Nutrient Mixture F-12 Ham complemented with 15 mM HEPES and sodium bicarbonate without L-glutamine (#D6421), insulin-transferrin-selenium liquid media supplement (#I3146), and 10% fetal bovine serum. The cells were maintained at 37°C with 5% CO_2_, and were ready for experiments. All media and supplements in this study were Sigma-Aldrich^®^ reagents (Merck KGaA, Germany).

### 2.2 Silencing of *CYP11B2* mRNA expression

Cells were silenced using either the Silencer Select Negative Control siRNA #2 (siScrambled) as control, or Silencer Select Pre-Designed siRNA CYP11B2 (siCYP11B2; Life Technologies Corporation, USA). Trypsinized HAC15 cells (1 × 10^6^) were transfected with 0.2 µM of siScrambled or siCYP11B2 using the Neon™ Transfection System (Life Technologies Corporation, USA). Electroporation was performed according to the manufacturer’s recommendation with the optimized parameters of 1,400 V, 20 ms, and 1 pulse. Electroporated cells were cultured in six-well plates containing 2 mL of antibiotic free medium with serum supplements for 24 h and then serum deprived in 1 mL of un-supplemented medium for 24 h. Supernatants were collected for aldosterone and cortisol measurements before proceeding to the apoptosis assay and/or cell RNA extraction.

### 2.3 Pharmacological inhibition of CYP11B2

Baxdrostat is a selective inhibitor of aldosterone synthase (CYP11B2), developed to suppress aldosterone biosynthesis while sparing cortisol production mediated by CYP11B1 (7, 13). In this study, HAC15 cells were treated with 10 ng/mL of baxdrostat (approximately 20–25 nM), a concentration selected based on the compound’s reported high potency and selectivity (14).

Trypsinized HAC15 cells were plated in six-well plates (at a density of 1 × 10^6^ cells/well) containing 2 mL of complemented medium. After 24 h, media was replaced with un-supplemented medium and cells were treated with either (i) 10 ng/mL baxdrostat (MedChemExpress, USA), (ii) 1:1,000 DMSO (Sigma-Aldrich, USA); (iii) 1:100 DMSO, or (iv) no treatment for 24 h. Supernatants were then collected for aldosterone and cortisol measurements before proceeding to the apoptosis assay and/or cell RNA extraction. Baxdrostat stock solution (10 µg/mL) was made using DMSO as the diluent whereby the final concentration of DMSO within the treatment media was 1:1,000.

### 2.4 Cell RNA extraction

Cells were kept in RNA*later*^®^ RNA Stabilization Reagent (Ambion, USA) according to the manufacturer’s recommendation until processed for RNA extraction. Total DNA-free RNA from cells was isolated using the PureLink™ RNA Mini Kit (Life Technologies Corporation, USA) in accordance with the manufacturer’s instruction. Extracted RNA was quantified and stored at −80°C until further analysis.

### 2.5 Transcriptomic analysis by RNA-sequencing

Total RNA used for transcriptomic analysis was extracted from HAC15 cells transfected with siRNA targeting CYP11B2 or scrambled control siRNA, obtained from three independent experiments. RNA integrity was assessed prior to library preparation, and only samples with an RNA integrity number (RIN) ≥7.0 were used for sequencing.

cDNA library constructions were performed, and paired-end (PE100) reads were sequenced commercially using the Sequencing Platform DNBseq™ (Beijing Genomics Institute (BGI, https://www.bgi.com). Raw sequencing reads were subjected to quality control prior to downstream analysis. Clean raw sequences (Phred+33) were obtained through a series of data processing using the SOAPnuke software developed by the sequencing company (15). Reads were aligned to the human reference genome (GRCh38) using a standard splice-aware aligner. Gene-level counts were generated and normalized across samples.

Dr. Tom, a web-based solution designed by the BGI for the convenient analysis, visualization, and interpretation of RNA data, was used to identify differentially expressed genes (DEGs) and to perform the pathway analysis (https://biosys.bgi.com/#/report/login). Differential expression analysis was performed by aligning clean reads to the reference genome (GCF_000001405.38_GRCh38.p12) and applying a threshold of |log_2_FC| > 1 with an adjusted *p*-value (*Q*-value) <0.05 to identify significantly DEGs. All Dr. Tom analytical parameters, including the specific algorithms and threshold settings used, are detailed in **Supplementary Table S1**. Given that RNA samples were derived from three independent biological experiments, differential-expression and pathway enrichment analyses were performed separately for each experiment. The resulting DEG lists were subsequently compared across experiments to assess reproducibility and identify genes and pathways consistently represented across independent experiments.

### 2.6 Effect of SB203580 and U0126 on adrenocortical hormones

The effect of SB203580, a p38 MAPK inhibitor, and U0126, a MEK1/2 inhibitor, was investigated on HAC15 cells steroidogenic output at concentrations previously reported to induce (1 µM SB203580) or inhibit autophagy (25–50 µM U0126), respectively (16–19). A total of 2.5 × 10^5^ HAC15 cells were randomly seeded in 24-well plates with 1 mL of un-supplemented medium. Forty-eight hours post-seeding, cells were treated with either (i) 1 µM SB203580 (InvivoGen, USA); (ii) 1, 10, 25, 50, or 100 µM U0126 (InvivoGen, USA); (iii) 400 µg/mL of G-418 (Gibco Life Technologies Corporation, USA), a protein synthesis and cell growth inhibitor; or (iv) 1 × 10^−6^ M AngII (Sigma-Aldrich, USA), an aldosterone stimulator and CYP11B2 inducer. After 24 h of treatments, the supernatants were harvested for aldosterone and cortisol measurements, and the cells were harvested for RNA isolation. Stock solutions of both SB203580 and U0126 were made with DMSO. Thus, all cells investigated were maintained in the presence of 1.04:1,000 DMSO.

### 2.7 EZMTT proliferation assay

Cell proliferation was assessed using the EZMTT™ Cell Proliferation Assay. A total of 2.5 × 10^3^ HAC15 cells were randomly seeded in 96-well plates in 100 µL of antibiotic-free medium with serum and supplements, and transfected with 0.03 µM of siScrambled or siCYP11B2 using the Lipofectamine 3000 Transfection Reagent (Life Technologies Corporation, USA). Seventy-two hours post-transfection, the proliferation assay was performed on transfected cells cultured in 100 µL of un-supplemented medium using the EZMTT Cell Proliferation Assay (Merck KGaA, Germany), with a final concentration of 1× EZMTT solution and in the presence of either (i) 400 µg/mL of G418 or (ii) 1 × 10^−6^ M AngII, or no additions. The cells’ proliferation rate was measured using the microplate reader at an absorbance 450 nm at 4, 24, 48, 72, and 96 h post-EZMTT addition timepoint.

### 2.8 Effect of SB203580 and U0126 on EZMTT assay

A total of 2.5 × 10^3^ HAC15 cells were seeded in 96-well plates and (i) transfected as mentioned in the EZMTT Proliferation Assay methodology previously described and cultured for 72 h, or (ii) 72 h post-seeding, treated with either 10 ng/mL baxdrostat or 1:1,000 DMSO as diluent control. The proliferation assay was then performed on transfected cells or baxdrostat/DMSO-treated cells cultured in 100 µL of un-supplemented medium with a final concentration of 1× EZMTT solution and with treatment with either (i) 1 µM SB203580; (ii) 25 µM or 50 µM U0126; (iii) 400 µg/mL of G418; or (iv) 1 × 10^−6^ M AngII. The cells’ proliferation rate was measured using the microplate reader at an absorbance of 450 nm at 4, 24, 48, 72, and 96 h post-EZMTT addition timepoint. All experimental conditions continued the same final concentration of DMSO (1.04:1,000).

### 2.9 Effect of SB203580 and U0126 on xCELLigence real-time cell analyzer

A total of 2.5 × 10^3^ or 5.0 × 10^3^ HAC15 cells were randomly seeded in a 96-well plate xCELLigence E-plate (#300601010, Agilent Technologies, USA). Forty-eight hours post-seeding, the cells were treated with either 10 ng/mL baxdrostat or 1:1,000 DMSO in 200 µL of un-supplemented medium with either (i) 1 µM SB203580; (ii) 25 or 50 µM U0126; (iii) 400 µg/mL of G418; or (iv) 1 × 10^−6^ M AngII. The impedance values were measured on xCELLigence real-time cell analyzer (RTCA) (#00380601030, Agilent Technologies, USA) at an hour interval for 96 h.

### 2.10 Flow cytometry apoptosis assay

Apoptosis assay was performed using the Pacific Blue™ Annexin V/SYTOX™ AADvanced™ Apoptosis Kit (Life Technologies Corporation, USA) in accordance with the manufacturer’s recommendation, after 48 h post-transfection of siRNAs or 24 h post-treatments. Labeled cell populations were then detected on a FACSVerse or FACSMelody with batch controls in all experiments (BD Biosciences, USA). Data were analyzed using FlowJo (version 10.8.1). Experiments were repeated three times independently in triplicates, with a minimum of 10,000 cells analyzed per sample.

### 2.11 Aldosterone and cortisol measurements

Aldosterone in the supernatant of the cells was measured on the fully automated Immunochemiluminescent IDS-iSYS platform (#IS-3300, Immunodiagnostic System UK). The Cobas e411 electrochemiluminescence immunoassay analyzer (#06687733190, Roche Diagnostics, Switzerland) was used to measure cortisol level in the cell medium. Protein concentration in each well, representing total amount of cells, was measured using the Bicinchoninic Acid Protein Assay Kit (Sigma-Aldrich, USA). The aldosterone and cortisol measured in the supernatant were normalized to the total cell protein in each well.

### 2.12 Gene expression validation by RT-PCR

The GoScript™ Reverse Transcription System, Promega (Promega Corporation, Wisconsin, USA), was used with a 1:1 mixture of random primer and oligodeoxythymidine (oligo dt) primers to synthesize the cDNA from a total of 1 µg of total cell RNA. mRNA expression of genes of interest and housekeeping gene was quantified using commercial TaqMan probes and the ABI Prism 7000 Sequence Detection Systems (Applied Biosystems, USA) as listed in **Supplementary Table S2**. Results were analyzed using the 2^−ΔΔCT^ relative quantitative method.

### 2.13 Western blot

HAC15 cells transfected with siCYP11B2 or siScrambled were lysed and immune-blotted using the following primary antibodies: CYP11B2 mouse monoclonal antibody (dilution 1:1,000; produced by Prof. Celso E. Gomez-Sanchez) or an anti-GAPDH mouse monoclonal antibody (dilution 1:5,000; #G8795, Sigma-Aldrich, USA). Secondary antibody used was the anti-Mouse IgG (Fab specific)–Peroxidase antibody produced in goat (dilution 1:10,000; #3682, Sigma-Aldrich, USA). Similarly, for pharmacological experiments, cells were subsequently lysed and CYP11B2 protein expression was assessed by Western blotting as described above.

### 2.14 Statistical analysis

Each experiment was performed at least three times independently in which each treatment/transfection was performed at least in triplicates. All results were derived from three independent biological experiments. All the results were expressed as mean ± standard error of the mean (SEM) unless otherwise indicated. Normal distribution of the data was determined using the Shapiro–Wilk statistical test. The difference between two groups with normal distributed data was analyzed for significance using the Student *t*-test whereas non-normally distributed data were analyzed using the Mann–Whitney *U* test. For multiple-group comparison, one-way or two-way ANOVA followed by Tukey *post-hoc* test was performed for normally distributed data or Kruskal–Wallis test followed by Dunn’s test for non-normally distributed data. Repeated measures were analyzed for significance using the two-way repeated measures ANOVA. Statistical significance was calculated using biological replicates, with technical replicates averaged within each experiment unless otherwise stated. The differences between groups were considered statistically significant if *p* < 0.05. Statistical analysis was performed using SPSS 29.0 for Windows (SPSS Inc., USA).

## 3 Results

### 3.1 Silencing of *CYP11B2* mRNA expression in human adrenocortical cells (HAC15)

Neon transfection of HAC15 cells with siCYP11B2 decreased expression of *CYP11B2* mRNA in siCYP11B2 cells by 88% ± 10.8% of siScrambled cells level (*n* = 9, *p* < 0.001, **Figure 1A**). The aldosterone production was also reduced by 70% ± 2.9% compared to transfected HAC15 siScrambled cells (*n* = 18, *p* < 0.001, **Figure 1B**). No differences in *CYP11B1* mRNA expression or cortisol production were detected (**Supplementary Figure S1**).

**Figure 1:**
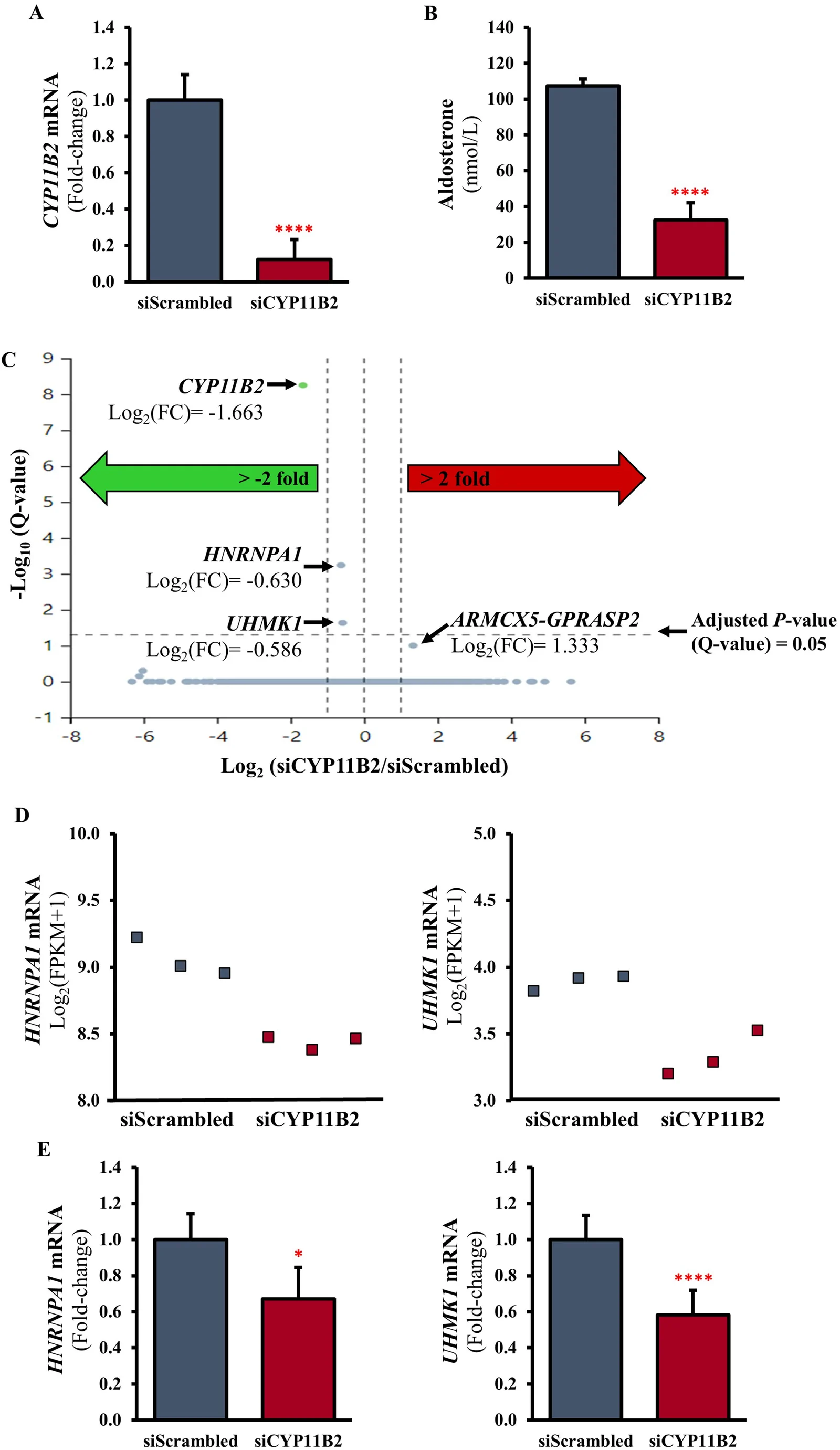
Silencing of *CYP11B2* reduced aldosterone production and downregulated *CYP11B2*, *HNRNPA1*, and *UHMK1* mRNA expression. **(A)** *CYP11B2* mRNA expression (fold change = 2^−ΔΔCT^) of siCYP11B2 cells compared to siScrambled cells (*n* = 9). **(B)** Aldosterone measurements of supernatant collected from a six-well plate for 24 h from HAC15 cells post-transfection with siCYP11B2 or siScrambled siRNA using the neon transfection system (*n* = 18). The aldosterone measurements were not normalized to the total cell protein in each well. **(C)** Volcano plot of DEGs [|log2FC| > 1, the adjusted *p*-value (*Q*-value) <0.05]. Genes that are downregulated are shown in green (siScrambled vs. siCYP11B2; *n* = 3 each a paired independent experiment). **(D)** *HNRNPA1* and *UHMK1* mRNA expression in siCYP11B2 cells and siScrambled transfected HAC15 cells as measured by RNA-seq. Data presented as log 2 of fragments per kilobase of transcript per million fragments mapped (FPKM) plus 1. **(E)** *HNRNPA1* and *UHMK1* mRNA expression in siCYP11B2 and siScrambled transfected HAC15 cells as measured by RT-PCR (*n* = 9). Data presented as geomean of fold-change (2^−ΔΔCT^) ± SEM. *p < 0.05; ****p < 0.001; compared to the siScrambled group. Statistical significance was calculated using the Student *t*-test.

RNA-sequencing (RNA-seq) of siCYP11B2 and siScrambled cells detected *HNRNPA1* and *UHMK1* mRNA expression to be consistently downregulated in all siCYP11B2 cells compared to siScrambled cells (**Figures 1C, D**). This was verified by RT-PCR (*HNRNPA1* by 33% ± 17.6%, *p* = 0.02; and *UHMK1* by 42% ± 13.7%*, n* = 9, *p* < 0.001; **Figure 1E**). *ARMCX5-GPRASP2* was the only gene found to be consistently upregulated in the siCYP11B2 group; however, this was not confirmed as statistically significant by RT-PCR (**Figure 1C**; **Supplementary Figure S2**). Additionally, there were no significant changes in key steroidogenesis genes (*STAR*, *CYP11A1*, *CYP17A1*, *HSD3B2*, and *CYP21A2*) in siCYP11B2 cells compared to siScrambled (**Supplementary Figure S3**). Despite the limited number of consistently regulated genes, pathway-level analysis performed separately for each of the three independent experiments identified autophagy- and mitophagy-related KEGG pathways among the pathways represented by DEGs. DEG analysis was based on an absolute log2 fold change >1 and adjusted *p*-value (*Q*-value) <0.05. The DEG and pathway-level results were subsequently compared across independent experiments to assess reproducibility and identify pathways consistently represented across experiments (**Figure 2**; **Supplementary Table S3**; **Supplementary Data File 1**).

**Figure 2:**
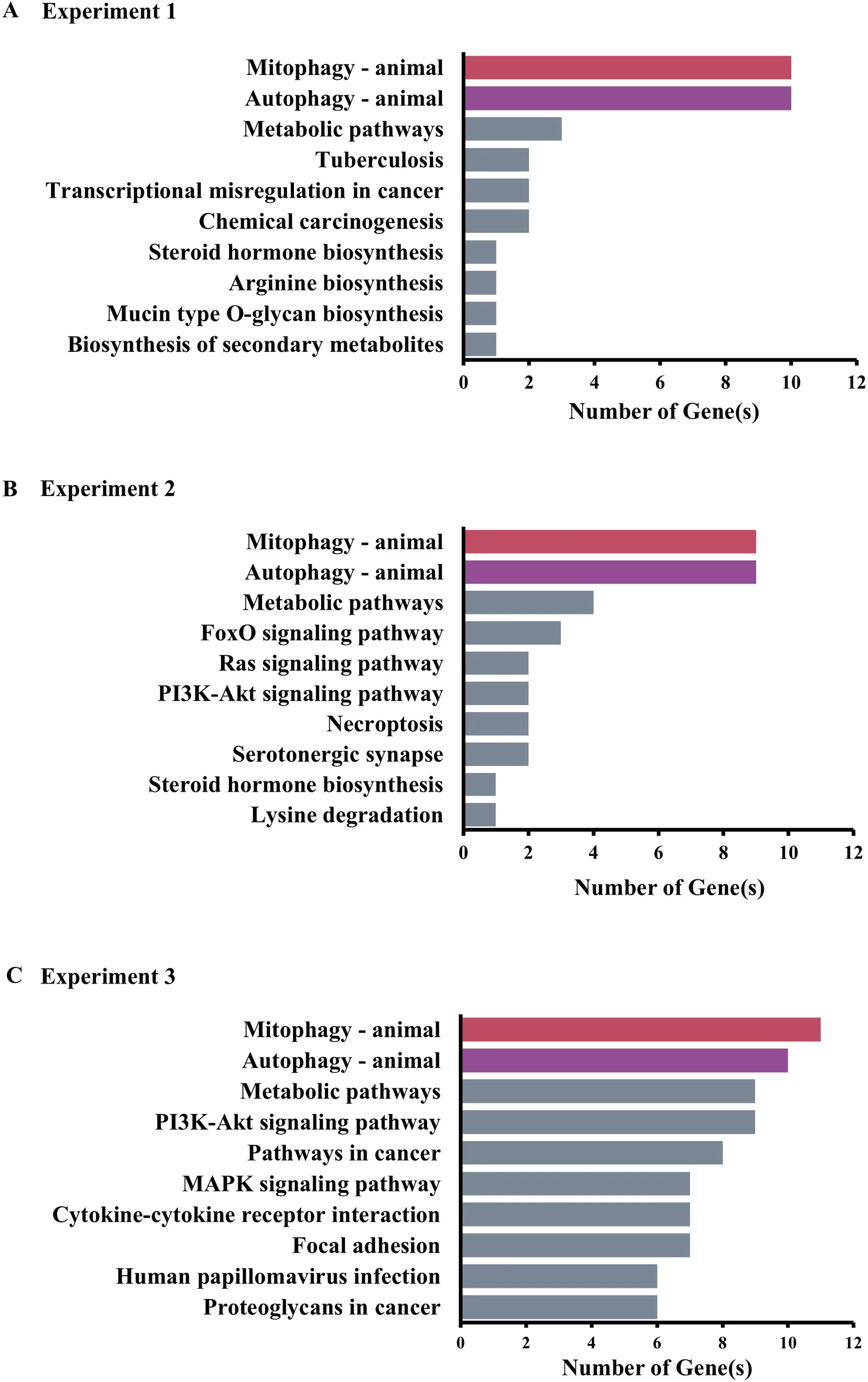
Mitophagy and autophagy were the top two pathways with most number of associated DEGs for siCYP11B2/siScrambled transfected cells. Top 10 KEGG pathway classification with most differentially expressed genes (DEGs) comparing siCYP11B2-transfected cells with siScrambled transfected cells from three independent experiments **(A–C)**.

### 3.2 Effects of SB203580 and U0126 on aldosterone and cortisol production

As autophagy- and mitophagy-related pathways were among the pathways containing the greatest numbers of DEG when comparing siScrambled and siCYP11B2 cells, the effect of modulating autophagy indirectly using SB203580 (p38 MAPK inhibitor) and U0126 (MEK1/2 inhibitor) was investigated on HAC15 cells’ steroidogenic output.

Treatment of 1 µM SB203580 increased aldosterone levels in HAC15 cells by 3.2 ± 0.5-fold (*n* = 11–12, *p* = 0.046; **Figure 3A**) without significantly affecting cortisol production (**Figure 3B**). This resulted in an increased aldosterone-to-cortisol ratio (4.5 ± 0.5-fold, *n* = 12, *p* < 0.001).

**Figure 3:**
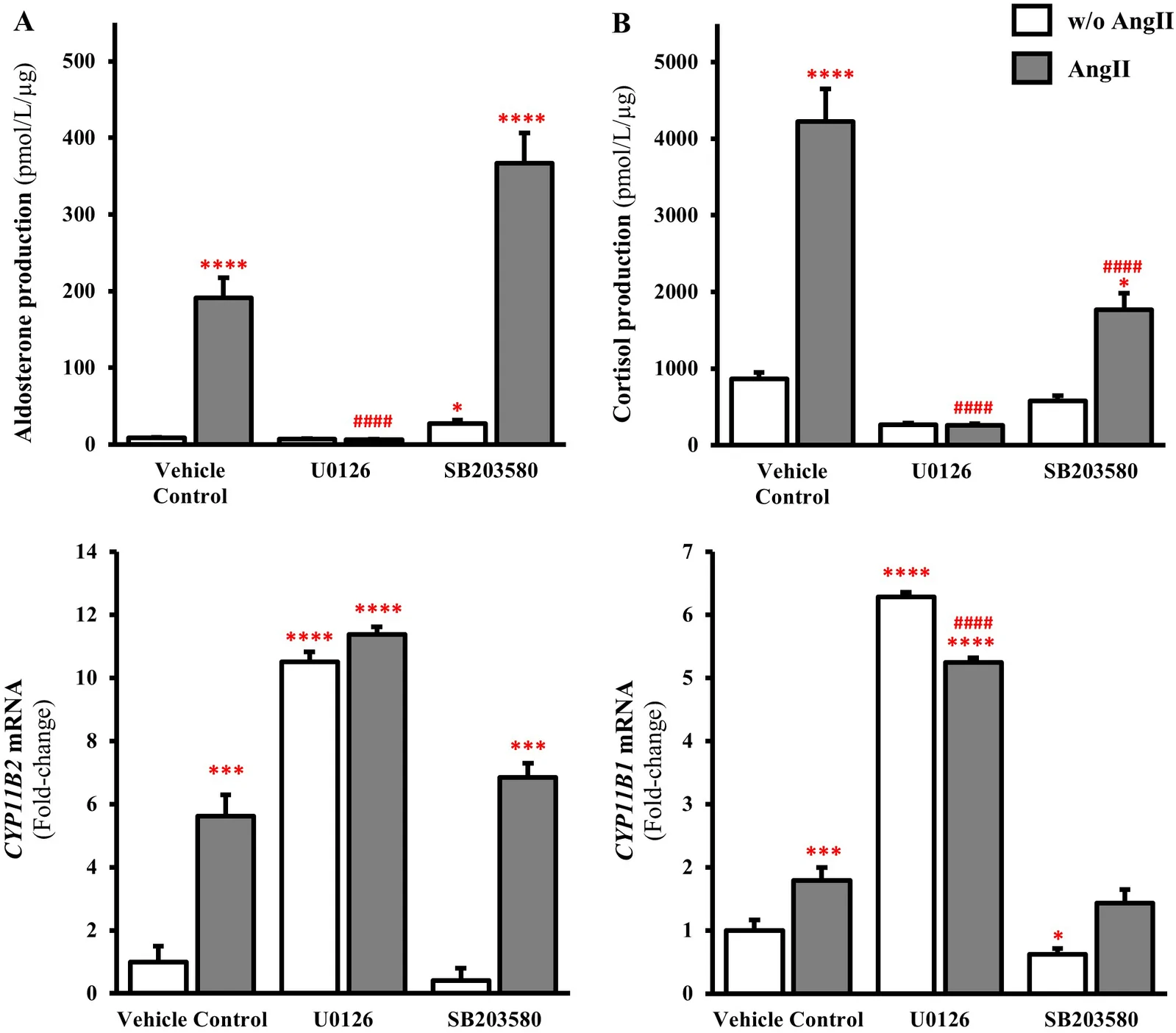
Effect of 25 μM U0126 or 1 µM SB203580 in HAC15 cells with or without (w/o) 1 µM AngII treatment. **(A)** Twenty-four-hour aldosterone production of treated cells from supernatant of 24-well plates (*n* = 11 for the vehicle control group, *n* = 12 for treatment groups), and *CYP11B2* mRNA expression from the cell extracts (*n* = 9). mRNA data presented as fold-change geomean (2^−ΔΔCT^) ± SEM. Statistical significance was calculated using Kruskal–Wallis followed by Dunn’s test. **(B)** Twenty-four-hour cortisol production of treated cells from supernatant of 24-well plates (*n* = 11 for the vehicle control group, *n* = 12 for treatment groups), and *CYP11B1* mRNA expression from the cell extracts (*n* = 9). mRNA data presented fold-change geomean (2^−ΔΔCT^) ± SEM. Statistical significance was calculated using one-way ANOVA followed by Tukey *post-hoc* test. w/o, without. *p < 0.05; ***p < 0.005; ****p < 0.001; compared to the vehicle control w/o AngII group. ^####^*p* < 0.001; compared to the vehicle control with AngII group.

Conversely, treatment with 1–100 µM U0126 reduced AngII-stimulated aldosterone and cortisol production in a dose-dependent manner (*n* = 8, *p* < 0.001; **Supplementary Figure S4A**). However, the effects of U0126 on *CYP11B2* and *CYP11B1* mRNA expression were variable (**Supplementary Figure S4B**). Notably, treatment with 25 µM U0126 suppressed aldosterone and cortisol production to levels comparable with unstimulated cells, despite markedly increased *CYP11B2* mRNA expression (11.38 ± 0.23-fold relative to untreated cells; *n* = 9, *p* < 0.001; **Figure 3A**) and *CYP11B1* mRNA expression (6.28 ± 0.08-fold change of untreated cells; *n* = 9, *p* < 0.001; **Figure 3B**). A similar unpairing of steroid production and mRNA expression was also observed in unstimulated cells, where 25 µM U0126 increased *CYP11B2* and *CYP11B1* expression (8.74 ± 0.26-fold, *p* < 0.01 and 4.34 ± 0.56-fold, *p* < 0.05, respectively) without significantly affecting aldosterone or cortisol production (**Supplementary Figure S4B**). Cell morphology and adherence were maintained at this concentration, suggesting that the reduction in hormone output was not unlikely to be attributable to overt cytotoxicity (**Supplementary Figure S4C**).

Comparable effects were observed following treatment with 50 µM U0126 (**Supplementary Figures S5A, B**). The effects of 25 µM U0126 and 50 µM U0126 are thought to reflect autophagy inhibition, as 40 μM U0126 pretreatment reduces the levels of LC3-II and Beclin-1 autophagy molecules induced by cisplatin in HEI-OC1 cells and cochlear hair cells (18). In addition, the increase in aldosterone production observed following SB203580 was negated by co-treatment with U0126 (**Supplementary Figure S6**).

Interestingly, the increase in AngII-stimulated aldosterone production following SB203580 treatment occurred without a corresponding increase in *CYP11B2* mRNA expression but was accompanied by increased CYP11B2 protein expression (3.50 ± 1.038 vs. 1.83 ± 0.636, *n* = 3, *p* = 0.049; **Figure 3**; **Supplementary Figure S6**; **Supplementary Table S4**). Similarly, despite the marked increase in *CYP11B2* mRNA expression following treatment with 25 and 50 µM U0126, CYP11B2 protein expression was not significantly altered (**Supplementary Figure S6**; **Supplementary Table S4**).

### 3.3 Effect of siCYP11B2 transfection on EZMTT absorbance

Using the EZMTT assay, an increase in absorbance was detected in the siCYP11B2-transfected cells compared to siScrambled cells (absorbance at 450 nm: 0.141 ± 0.02 AU at the 96-hour timepoint, *n* = 90, *p* < 0.001, **Figure 4A**). The controls, G418, a protein synthesis and cell growth inhibitor, and AngII, an aldosterone stimulator and CYP11B2 inducer, decreased or increased absorbance respectively in both transfected cells (*n* = 35, *p* < 0.001; **Figure 4B**). Although no difference in aldosterone production was observed between siCYP11B2- and siScrambled-transfected cells in the 96-h accumulated supernatants (FC = 0.78 ± 0.134, *n* = 15, *p* = 0.15, **Figure 4C**), less protein expression of CYP11B2 (0.3-fold change) was seen by Western blot in siCYP11B2-transfected cells compared to siScrambled cells at 72 h post-transfection (**Figure 4D**). To note, the transient effect of the transfection could have worn off 168 h post-transfection (72 h + 96 h).

**Figure 4:**
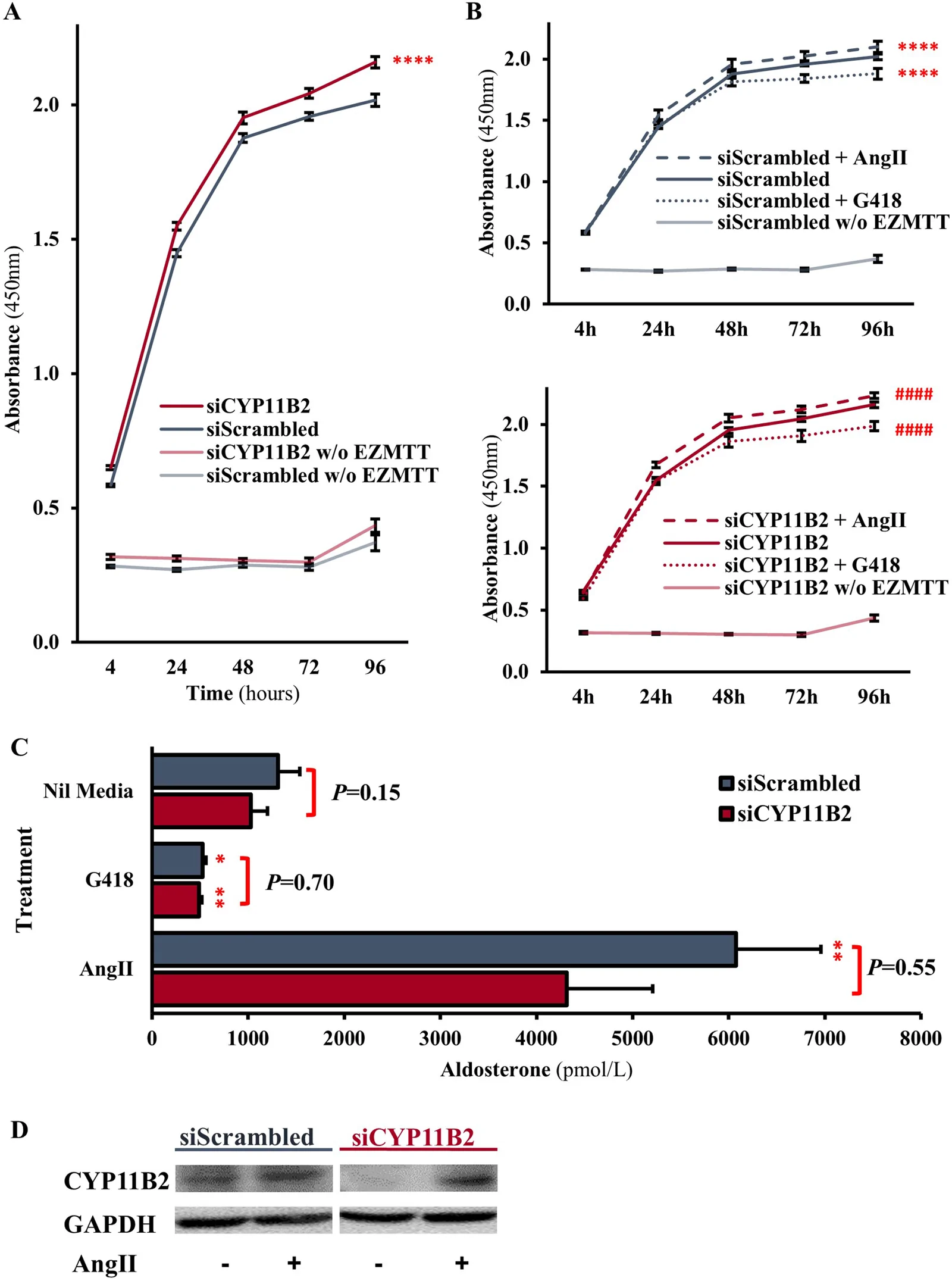
EZMTT assay detected an increase in absorbance in siCYP11B2-transfected cells. **(A)** Absorbance measurements in siCYP11B2 and siScrambled (*n* = 90) cells treated with EZMTT 72 h post-transfection, measured at 4, 24, 48, 72, and 96 h post-EZMTT addition. Statistical significance was calculated using the two-way repeated measures ANOVA test. **(B)** Absorbance measurements in siCYP11B2 and siScrambled cells treated with G418 or AngII (*n* = 35) and EZMTT 72 h post-transfection, measured at 4, 24, 48, 72, and 96 h post-EZMTT addition. Statistical significance was calculated using the two-way repeated measures ANOVA test. **(C)** Aldosterone measurements of supernatant collected from 96-well plates at Day 7 post-EZMTT (*n* = 6–15). Statistical significance was calculated using Kruskal–Wallis followed by Dunn’s test. The aldosterone measurements were not normalized to the total cell protein in each well. **(D)** Western blot protein expression of HAC15 cells 72 h post-transfection with siCYP11B2 or siScrambled in the absence or presence of 24 h AngII treatment. siCYP11B2 cells had 0.3-fold CYP11B2 protein expression of siScrambled cells (normalized by GAPDH). w/o, without. *p < 0.05; **p < 0.01; ****p < 0.001; compared to the siScrambled group. ^####^*p* < 0.001; compared to the siCYP11B2 group.

### 3.4 Effect of SB203580, U0126, and CYP11B2 inhibitor on EZMTT absorbance and xCELLigence RTCA cell index

In separate siCYP11B2 and siScrambled-transfected cells, SB203580 did not increase the EZMTT assay absorbance whereas a substantial decrease of absorbance was seen with U0126 treatment (FC = 0.68 ± 0.043 and FC = 0.73 ± 0.048, respectively; *n* = 25, *p* < 0.001; **Figure 5A**). No differences were seen with 10 ng/mL baxdrostat treatment compared to vehicle control, for EZMTT absorbance (**Figure 5B**) or for the area under the curve (AUC) of xCELLigence RTCA cell index—both at lower density of cells (2,500 cells) or at higher density of cells (5,000 cells; **Supplementary Figure S7A**). Effects of U0126, AngII, and G418 seen with EZMTT were reciprocated with the AUC of xCELLigence RTCA cell index (**Supplementary Figure S7B**).

**Figure 5:**
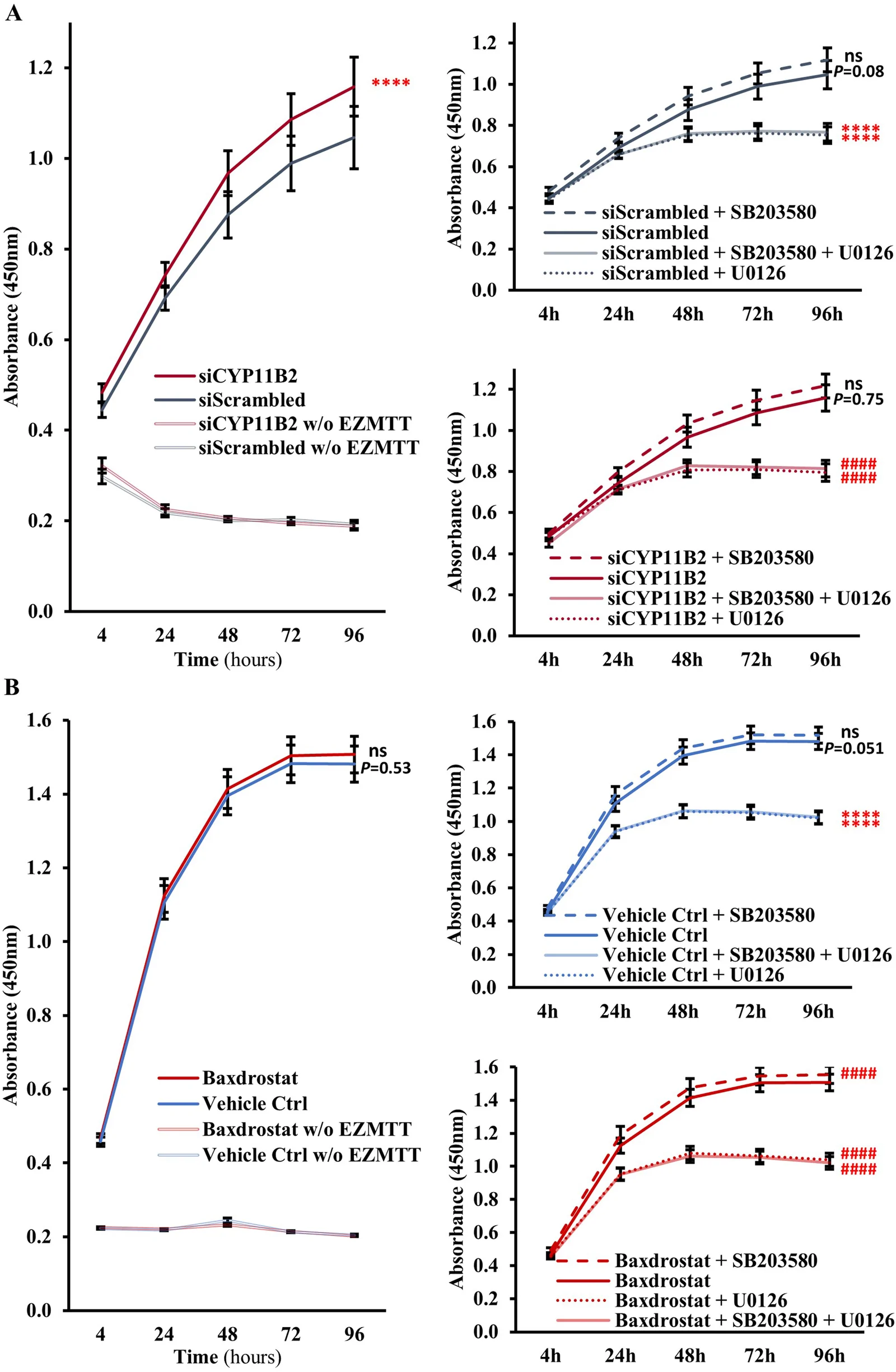
Cell proliferation effects of U0126 and SB203580 on siCYP11B2-transfected or baxdrostat-treated HAC15 cells. **(A)** Absorbance measurements in siCYP11B2 and siScrambled cells treated with 1 µM of SB203580 or 25 µM of U0126 and EZMTT 72 h post-transfection, measured at 4, 24, 48, 72, and 96 h post-EZMTT addition (*n* = 25). **(B)** Absorbance measurements in baxdrostat- and DMSO 1:1,000 (vehicle control)-treated cells in the presence of 1 µM of SB203580 or 50 µM of U0126 and EZMTT 72 h post-seeding, measured at 4, 24, 48, 72, and 96 h post-EZMTT addition (*n* = 45). w/o, without. ns, not significant; compared to the siScrambled group, the siCYP11B2 group, or the vehicle control group. ****p < 0.001; compared to the siScrambled group or the vehicle control group. ^####^*p* < 0.001; compared to the siCYP11B2 group or the baxdrostat group. Statistical significance was calculated using a two-way repeated measures ANOVA test.

### 3.5 Effect of CYP11B2 modulation on HAC15 cell apoptosis

Flow cytometry analysis of siCYP11B2-transfected cells showed no significant differences in the fold change of viable, early apoptotic, late apoptotic, or necrotic cell populations compared with siScrambled controls (*n* = 12, **Figure 6A**; **Supplementary Figure S8**).

**Figure 6:**
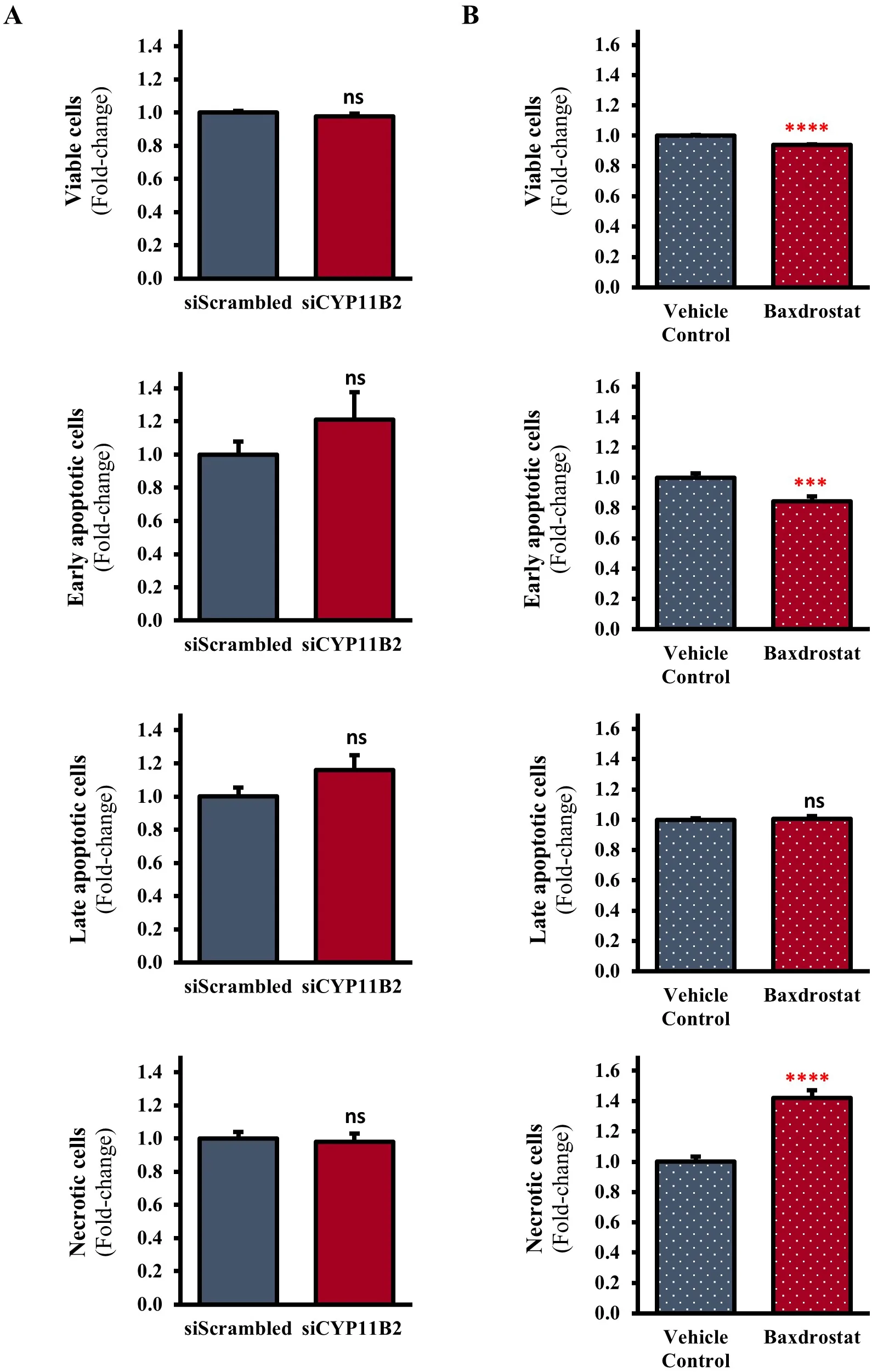
Silencing of CYP11B2 has no effect on cell apoptosis, while pharmacological inhibition of the CYP11B2 using baxdrostat increased necrotic cells (FC = 1.42 ± 0.05, *n* = 144 and *p* = 1.3 × 10^−10^). **(A)** Flow cytometry measurements of viable, early apoptotic, late apoptotic, and necrotic HAC15 cells 48 h post-transfection with siCYP11B2 compared to siScrambled (*n* = 12). **(B)** Flow cytometry measurements of viable, early apoptotic, late apoptotic, and necrotic HAC15 cells 24 h post-treatment with baxdrostat compared to vehicle control (DMSO 1:1,000) (*n* = 144). ns, not significant; compared to the siScrambled group or the vehicle control group. ***p < 0.005; ****p < 0.001; compared to the siScrambled group or the vehicle control group. Statistical significance was calculated using the Mann–Whitney *U* test.

In contrast, pharmacological inhibition of CYP11B2 with the selective aldosterone synthase inhibitor baxdrostat was associated with altered distributions of viable and cell-death populations compared with vehicle controls across the pooled experiments (*n* = 144, **Figure 6B**). However, the relative distribution of apoptotic and necrotic populations varied between independent experiments (**Supplementary Figure S9**). Notably, Experiment 3 showed a significant increase in apoptotic cells (FC = 1.15 ± 0.04, *n* = 36, *p* = 9.1 × 10^−4^) without a corresponding increase in necrotic cells (**Supplementary Figures S9**, **S10A, B**).

A similar pattern was observed with the apoptosis-positive control, DMSO 1:100, compared with the vehicle control, DMSO 1:1,000, which showed a significant decrease in viable cells (FC = 0.9 ± 0.01, *n* = 12, *p* = 0.006) and an increase in apoptotic cells (FC = 1.2 ± 0.05, *n* = 12, *p* = 0.041), with no significant change in necrotic cells (**Supplementary Figure S10C**).

The 10 ng/mL baxdrostat treatment selectively reduced aldosterone production compared with vehicle control (FC = 0.52 ± 0.03, *n* = 24, *p* = 3.5 × 10^−6^), without affecting cortisol production (**Supplementary Figures S11A, B**). No significant CYP11B2 expression was detected under these experimental conditions. Total cellular protein content did not differ between treatment groups, suggesting that the observed changes in cell populations were not associated with an overall reduction in cellular biomass and may instead reflect altered cell turnover (**Supplementary Figure S11C**).

## 4 Discussion

In this study using HAC15 human adrenocortical cells, reduction of CYP11B2 expression decreased aldosterone production without inducing consistent changes in apoptosis, but was associated with transcriptional signatures enriched for autophagy and mitophagy pathways. These findings suggest that steroidogenic capacity is linked to cellular processes involved in mitochondrial quality control and intracellular homeostasis.

Selective silencing of *CYP11B2* reduced aldosterone production by approximately 70% without affecting *CYP11B1* expression or cortisol output, confirming specificity despite high sequence homology between enzymes. This was accompanied by downregulation of *HNRNPA1* and *UHMK1* mRNA expression and a trend toward upregulation of *ARMCX5-GPRASP2* mRNA. *HNRNPA1*, encoding the heterogeneous nuclear ribonucleoprotein A1 protein, and *UHMK1*, encoding the U2AF homology motif kinase 1, are both involved in the splicing process (20, 21). Previous studies have shown overexpression of *HNRNPA1* and *UHMK1* in several cancers: lung (22–24), breast (25), colon (26, 27), and hepatocellular cancer (28, 29). The proteins in the cancer tissues are thought to promote cell cycle progression and cell proliferation, and inhibit cell senescence, necroptosis, apoptosis, and autophagy, thus leading to acceleration of cancer progression, whereas the Armadillo repeat containing X-linked 5 (*ARMCX5*)–G protein-coupled receptor-associated sorting proteins 2 (*GPRASP2*) enables G protein-coupled receptor binding, though the actual role or function of this gene is not yet fully elucidated. However, *ARMCX5-GPRASP2* expression has also been associated with autophagy and mitophagy (30–32).

Although relatively few genes were consistently differentially expressed across experiments, pathway-level analyses identified enrichment of genes related to autophagy and mitophagy processes. Variability in individual gene-level responses across the three independent biological experiments, together with differences in read abundance and dispersion, may account for the observation that some genes exhibited relatively large fold changes without reaching statistical significance. Autophagy is a protective mechanism crucial for maintaining cell function and cell survival under stress or nutrient deprivation by forming autolysosomes to degrade damaged or senescent cellular components for recycling (33, 34), whereas autophagy of degrading damaged mitochondria is called mitophagy (35). Autophagy is thought to prevent apoptosis, but can lead to a form of Type II programmed cell death when in excess or uncontrolled (36–38). Autophagy at a physiological level is hypothesized to enhance cell survival and, in excess, promote the degradation of essential cell viability component(s) that might induce cell death (39, 40). Thus, in certain conditions, autophagy promotes cell death. Beyond its degradative role, autophagy is also implicated in the regulation of the secretory pathway (41). In the inner ear, deficiency in autophagy disrupts the assembly and secretion of otoconial core proteins, thereby impairing otoconia development, leading to loss of balance in mice (42).

The association of *CYP11B2* silencing with autophagy and mitophagy is consistent with its role as a mitochondrial class I cytochrome P450 enzyme located in the inner mitochondrial membrane (43). To explore pathways potentially associated with the transcriptomics observations, the effects of indirect pharmacological modulators, U0126 and SB203580, on steroidogenic output were examined. Treatment with 25 µM of U0126 reduced aldosterone production to unstimulated levels despite a marked upregulation of *CYP11B2* mRNA expression (11.38 ± 0.23-fold). A similar dissociation between transcript abundance and hormone production was observed in unstimulated cells treated with U0126. Conversely, treatment with 1 µM SB203580 increased aldosterone production without altering *CYP11B2* mRNA expression. In contrast, changes in CYP11B2 protein expression appeared to correspond more closely with the direction of aldosterone production. Together, these findings demonstrate a dissociation between steroidogenic gene expression, CYP11B2 protein abundance, and steroid hormone production. These observations suggest that post-transcriptional mechanisms or other downstream processes may contribute to the regulation of aldosterone synthesis. However, the present experiments do not establish the precise mechanisms underlying these effects, including whether autophagy or mitophagy contributes directly to the observed changes.

Several mechanisms may account for the observed dissociation between steroidogenic gene expression and hormone production. For example, alterations in post-transcriptional regulation, protein translation or stability, mitochondrial function, or upstream steps in the steroidogenic pathway could limit steroid hormone synthesis despite preserved or increased CYP11B2 mRNA expression. Disruption of cholesterol transport or pregnenolone synthesis may also affect substrate availability for downstream steroidogenesis. Consistent with the potential importance of mitochondrial homeostasis in aldosterone-related pathophysiology, reductions in cardiac mtDNA and COX IV protein levels have been reported in aldosterone-infused ICR mice (44). This was accompanied by reduced ATP production, indicating compromised mitochondrial function. While these findings suggest that aldosterone may contribute to mitochondrial dysfunction, it remains unclear whether these effects are mediated directly through the mineralocorticoid receptor (MR), or indirectly through alternative pathways. Nonetheless, the observed mitochondrial impairment supports a role for mitophagy in maintaining the health and survival of aldosterone-producing cells, particularly under conditions of altered hormone synthesis capability although the relevance of these findings to the present HAC15 cell model remains to be established.

Autophagy also plays a role as a tumor suppressor by averting the activation of oncogenes in normal cells. *In vivo* animal research has shown that tumors develop when an autophagy gene, such as *Beclin1*, *Atg5*, or *Atg7*, is deleted (45–47). However, basal autophagy also promotes tumor growth and the advancement of cancer disease in established tumor cells or when an oncogene is activated. Interestingly, disruption of the autophagy gene Atg7 in the pancreatic β cells of mutant mice β cells leads to defective glucose regulation (48). These autophagy-deficient β cells had reduced survival, impaired insulin secretion, and abnormal organelle turnover, resulting in insulin deficiency and hyperglycemia. These findings highlight autophagy as a critical pathway for maintaining cell function and fate in an endocrine organ.

Our previous studies demonstrated that high expression of ZG-selective genes *LGR5* and *DACH1* inhibits aldosterone production and may increase apoptosis (10, 11). In non-human adrenal [mouse aldosterone synthase knock out (8) and monkey treated with aldosterone synthase inhibitor (7)], aldosterone production appears essential for ZG cell maintenance, as modulation was associated with ZG cell migration and apoptosis. We thus initially hypothesized that in physiological ZG cells (but not in autonomous aldosterone-producing cells harboring aldosterone-driving somatic mutations), selective ZG genes act as molecular “switches” that shift cell function from hormone synthesis to apoptosis/migration when aldosterone is not required—possibly as an adaptive response to the high-salt content of the modern diet (49). However, siCYP11B2 cells did not show consistent changes in cell apoptosis. Instead, EZMTT assays indicated increased cell proliferation, though the EZMTT assay does quantify cell proliferation by measuring the conversion of the tetrazolium salt MTT to formazan by active mitochondria (50). Hence, if the mitochondria are affected due to silencing of *CYP11B2*, the results may need to be interpreted with caution. Conversely, pharmacological inhibition of CYP11B2 using baxdrostat resulted in variable cell death as detected by SYTOX™ AADvanced™ staining, while no significant difference in the AUC of xCELLigence RTCA cell index was observed. However, the heterogeneity observed with baxdrostat (necrosis vs. apoptosis) requires further mechanistic dissection. The differing cellular phenotypes observed following CYP11B2 silencing and baxdrostat treatment may reflect the distinct mechanisms, kinetics, and duration of these interventions. siCYP11B2 reduces CYP11B2 expression, whereas baxdrostat inhibits CYP11B2 enzymatic activity. These approaches may therefore not produce equivalent cellular responses. In addition, compound-specific and vehicle-related effects cannot be completely excluded in pharmacological experiments. Nevertheless, in a study on 15 patients with PA treated with baxdrostat, there was persistent aldosterone suppression post-withdrawal, which supports a reduced population of active, functioning aldosterone-producing cells (51).

Several limitations of this study should be acknowledged. First, HAC15 cells, while widely used as a model of human adrenal ZG, are derived from an adrenocortical carcinoma and may not fully recapitulate the physiology of normal ZG cells (52, 53). Their neoplastic origin may also affect basal autophagy and mitophagy responses, thereby limiting generalizability. Thus, the observed increase in proliferation following *CYP11B2* silencing may reflect compensatory responses or limitations of the HAC15 model. Second, autophagic flux was not directly measured. The pharmacological agents used to modulate autophagy—U0126 and SB203580—are known to influence MAPK/ERK and p38 pathways, respectively, and may exert off-target effects independent of autophagic flux (54, 55). Furthermore, autophagic and mitophagic flux were not directly assessed in this study using LC3-II/LC3-I ratio, p62/SQSTM1 turnover, or lysosomal inhibition assays. Therefore, the identification of autophagy- and mitophagy-related transcriptomic pathways should be regarded as hypothesis-generating and does not establish functional activation or inhibition of these processes following CYP11B2 suppression. Further mechanistic studies will be required to determine whether these pathways contribute to the observed changes in steroidogenesis and cellular homeostasis. Third, our functional assays focused on terminal steroid hormone outputs (aldosterone and cortisol) and did not quantify upstream steroidogenic intermediates. Future studies measuring steroidogenic intermediates, including pregnenolone, progesterone, and deoxycorticosterone, together with steroidogenic acute regulatory (StAR) protein expression, may help identify the level within the steroidogenic cascade at which these effects occur. Finally, transcriptomic changes in genes such as *HNRNPA1* and *UHMK1* were not validated at the protein level. Further studies using primary ZG cells examining steroidogenic intermediates and the role of DEGs like *HNRNPA1* and *UHMK1*, with autophagy protein markers, would help validate the role of autophagy and mitophagy in ZG aldosterone regulation, cell survival, and metabolic state.

In summary, reduced aldosterone synthesis in HAC15 cells is associated with transcription signatures linked to autophagy and mitophagy, and pharmacological modulation of these pathways alters steroidogenic output independently of CYP11B2 expression. These findings support a model in which mitochondrial and autophagy-related processes contribute to the regulation of aldosterone biosynthesis and adrenal cell homeostasis. This raises the possibility that sustained inhibition of aldosterone synthase may exert previously unrecognized effects on adrenal cellular homeostasis beyond blood pressure control, warranting further mechanistic and *in vivo* investigation.

## 5 Perspective

In animal models, inhibition of aldosterone production is associated with apparent increased ZG cell turnover (proliferation, migration, and/or apoptosis), suggesting a dynamic relationship between steroidogenic activity and adrenal cortical homeostasis. In human adrenals, ZG-selective genes such as *LGR5* and *DACH1* have been linked to reduced aldosterone production and promotion of apoptotic pathways when steroidogenesis is not required. Together, these observations support the concept that ZG homeostasis is actively regulated in response to physiological demand.

From an evolutionary perspective, such adaptive mechanisms may have developed to maintain sodium balance under fluctuating dietary salt conditions. However, in the context of chronically high salt intake characteristic of modern diets, these regulatory processes may become maladaptive. One potential consequence is the selection or expansion of aldosterone-driver somatic mutations, contributing to the development of aldosterone-producing adenomas. The involvement of mitochondrial processes, including autophagy and mitophagy, in these adaptations warrants further investigation.

Further studies are required to define the mechanistic links between steroidogenic activity, cellular turnover, and adrenal zonation, and to determine how these processes contribute to the pathogenesis of PA.

## Data Availability

The datasets presented in this study can be found in online repositories. The names of the repository/repositories and accession number(s) can be found below: https://www.ncbi.nlm.nih.gov/, PRJNA1353593.
