# Supplementary material for: Selective inhibition of aldosterone production modulates human adrenocortical (HAC15) cell fate
Source: Front Endocrinol (Lausanne). 2026 Oct 12;17:1872432. doi: 10.3389/fendo.2026.1872432 (PMC13696181; doi:10.3389/fendo.2026.1872432)
Supplement: Supplementary file 1 [file DataSheet1.pdf]

SUPPLEMENTARY MATERIAL

Table S1. RNA-Seq analysis parameters

**Category**	**Parameter**	**Setting/Value**
**Quality Control**	Software	SOAPnuke v2.0
	Quality threshold	Phred score ≥ 20
	Read filtering	Remove adapters, low-quality reads
**Alignment**	Software	HISAT2 v2.1.0
	Reference genome	GRCh38 (GCF_000001405.38_GRCh38.p12)
	Alignment mode	Splice-aware, paired-end
**Quantification**	Software	featureCounts v2.0.1
	Annotation	GENCODE v35
	Count type	Gene-level counts
**Normalization**	Method	TPM (Transcripts Per Million)
	Also reported	FPKM (Fragments Per Kilobase per Million)
**Differential Expression**	Software	DESeq2
	Experimental design	~ Condition (siCYP11B2 vs. siScrambled)
	Fold change calculation	$\log_2$ ratio (siCYP11B2 / siScrambled)
	Significance threshold	$\lvert\log_2 FC\rvert > 1$
	P-value adjustment	Benjamini-Hochberg (FDR)
	Adjusted P-value threshold	< 0.05 (Q-value < 0.05)
**Pathway Enrichment**	Database	KEGG (Kyoto Encyclopedia of Genes and Genomes)
	Analysis method	Hypergeometric test / Over-representation analysis
	Background set	All genes in reference genome
	P-value adjustment	Benjamini-Hochberg (FDR)
	Significance threshold	Adjusted *P*-value < 0.05
	Ranking metric	Number of DEGs mapping to pathway
**Visualization**	Volcano plot	$-\log_{10}$(Q-value) vs. $\log_2 FC$
	Horizontal threshold	Q-value = 0.05 (i.e., $-\log_{10}(0.05) = 1.3$)
	Vertical thresholds	$\lvert\log_2 FC\rvert = 1$

Table S2. TaqMan gene expression assays used for validation

Gene	Applied Biosystem Assay ID	Reporter Dye
18s	Hs99999901_s1	VIC
ARMCX5-GPRASP2	Hs01005303_g1	FAM
CYP11B1	Hs01596406_m1	FAM
CYP11B2	Hs01597732_m1	FAM
GAPDH	Hs02786624_g1	VIC
HNRNPA1	Hs01656228_s1	FAM
UHMK1	Hs00332674_m1	FAM

Table S3. RNA-seq differentially expressed genes detected in each experiment related to autophagy and mitophagy pathways

Gene	Experiment No $P<0.05$	Log_2 [siCYP11B2 / siScrambled]	Overall *P*-value
ADAMTSL4	**3**	**-2.174**	**0.016**
ARMCX5-GPRASP2	**1, 2**	**1.333**	**<0.001**
ATG9B	1	1.969	0.052
C7orf55-LUC7L2	1, 2	-0.866	0.639
CLMP	3	-3.999	0.308
DNAJC25-GNG10	1	5.630	0.139
FAM156A	**1**	**0.758**	**0.035**
FRMD4A	3	-0.956	0.052
FSBP	1	0.650	0.682
HSFX2	3	-1.753	0.641
LDLRAD2	1, 2	-0.010	0.994
MXRA8	3	-0.386	0.239
NOMO3	1	-0.177	0.726
NPIPB13	2	-0.099	0.761
PHLDA3	3	-0.666	0.177
PHOSPHO2-KLHL23	2	0.634	0.852
PLEKHA6	**3**	**-2.678**	**0.036**
RAB34	3	-2.482	0.065
S100A11	**3**	**-3.061**	**0.048**
SERF1A	2	0.654	0.072
SERF1B	2	-0.353	0.138
SHISA2	3	-2.779	0.093
SRC	3	-0.740	0.297
TMEM110-MUSTN1	1	0.121	0.909
TVP23C-CDRT4	1, 2	0.568	0.223
ZNF765-ZNF761	2	0.084	0.857

Table S4. Effects of U0126 and SB203580 on AngII-stimulated CYP11B2 protein expression in HAC15 cells

	Fold-change over Vehicle Control			
	without AngII		AngII	
	**Average**	***P*-value***	**Average**	***P*-value***
Vehicle Control	1.00±0.000		1.83±0.636	ns
1 µM SB203580	1.03±0.191	ns	3.50±1.038	0.049
25µM U0126	0.51±0.146	ns	0.93±0.238	ns
50µM U0126	0.31±0.211	ns	0.34±0.165	ns
25µM U0126 + 1 µM SB203580	0.79±0.462	ns	1.11±0.482	ns
50µM U0126 + 1 µM SB203580	1.20±0.651	ns	1.16±0.580	ns

* Statistical significance was calculated using 1-way ANOVA followed by Tukey post hoc test. ns, not significant; compared to Vehicle Control group.

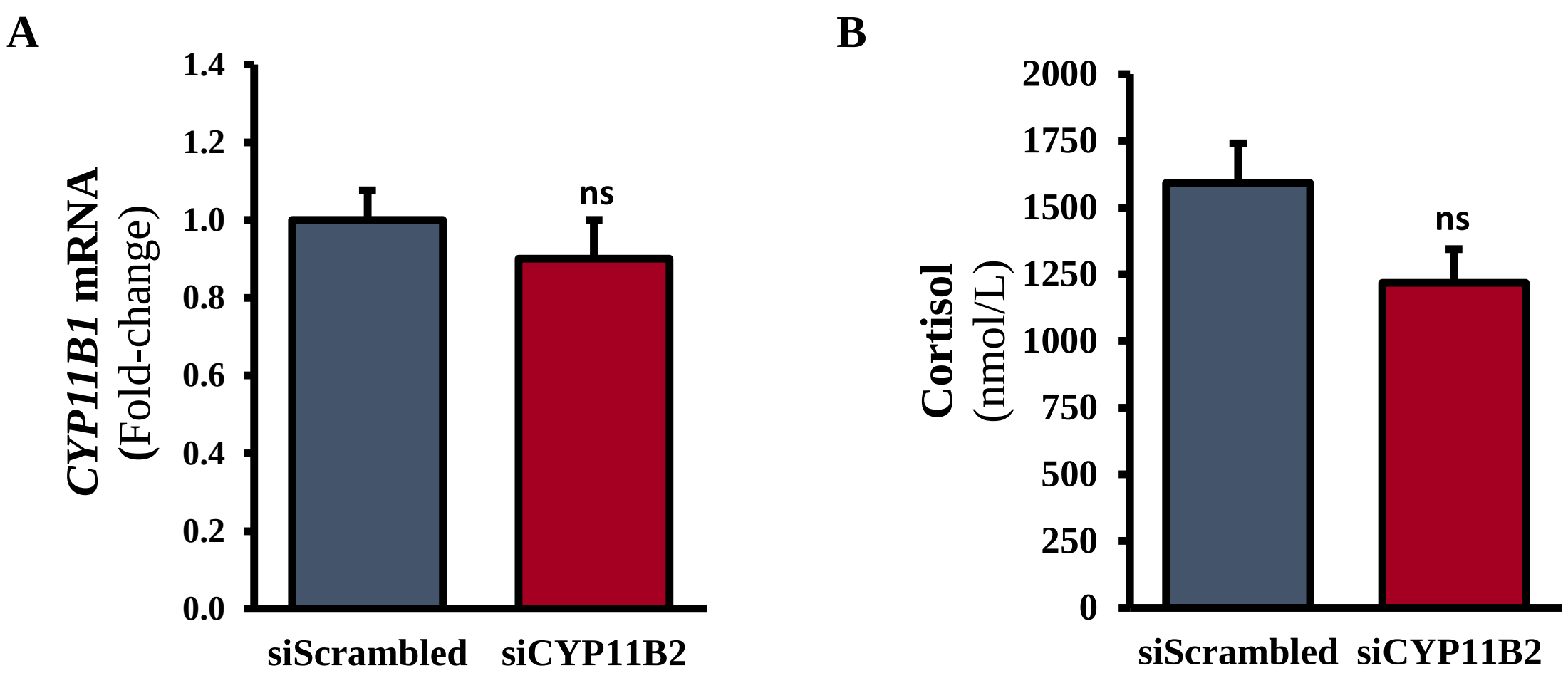

Figure S1. siCYP11B2 transfected cells showed no significant decrease in *CYP11B1* expression or cortisol production compared to siScrambled transfected cells.

(A) *CYP11B1* mRNA expression of siCYP11B2 cells compared to siScrambled cells (*n*=9). Data presented as geomean of fold-change ($2^{-\Delta\Delta CT}$)±SEM.

(B) 24 hours cortisol measurements of supernatant collected from 6 well plates from HAC15 cells post-transfection with siCYP11B2 or siScrambled siRNA using the neon transfection system (*n*=18). The cortisol measurements were not normalized to the total cell protein in each well.

ns, not significant; compared to siScrambled group.

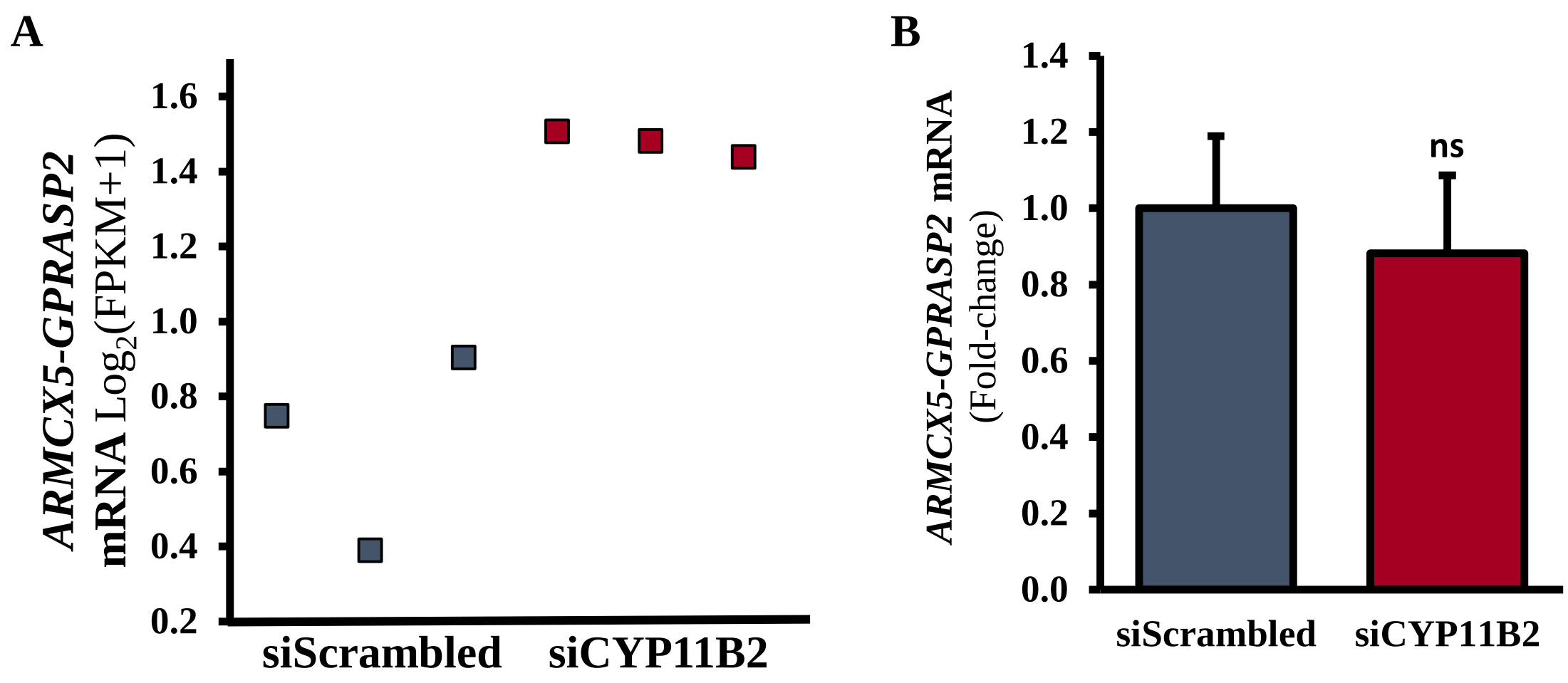

Figure S2. *ARMCX5-GPRASP2* expression in siCYP11B2 cells.

(A) *ARMCX5-GPRASP2* mRNA expression in siCYP11B2 cells and siScrambled transfected HAC15 cells as measured by RNA-seq. Data presented as log 2 of fragments per kilobase of transcript per million fragments mapped (FPKM) plus 1.

(B) *ARMCX5-GPRASP2* mRNA expression in siCYP11B2 and siScrambled transfected HAC15 cells as measured by RT-PCR (*n*=9). Data presented as geomean of fold-change ($2^{-\Delta\Delta CT}$)±SEM.

ns, not significant; compared to siScrambled group.

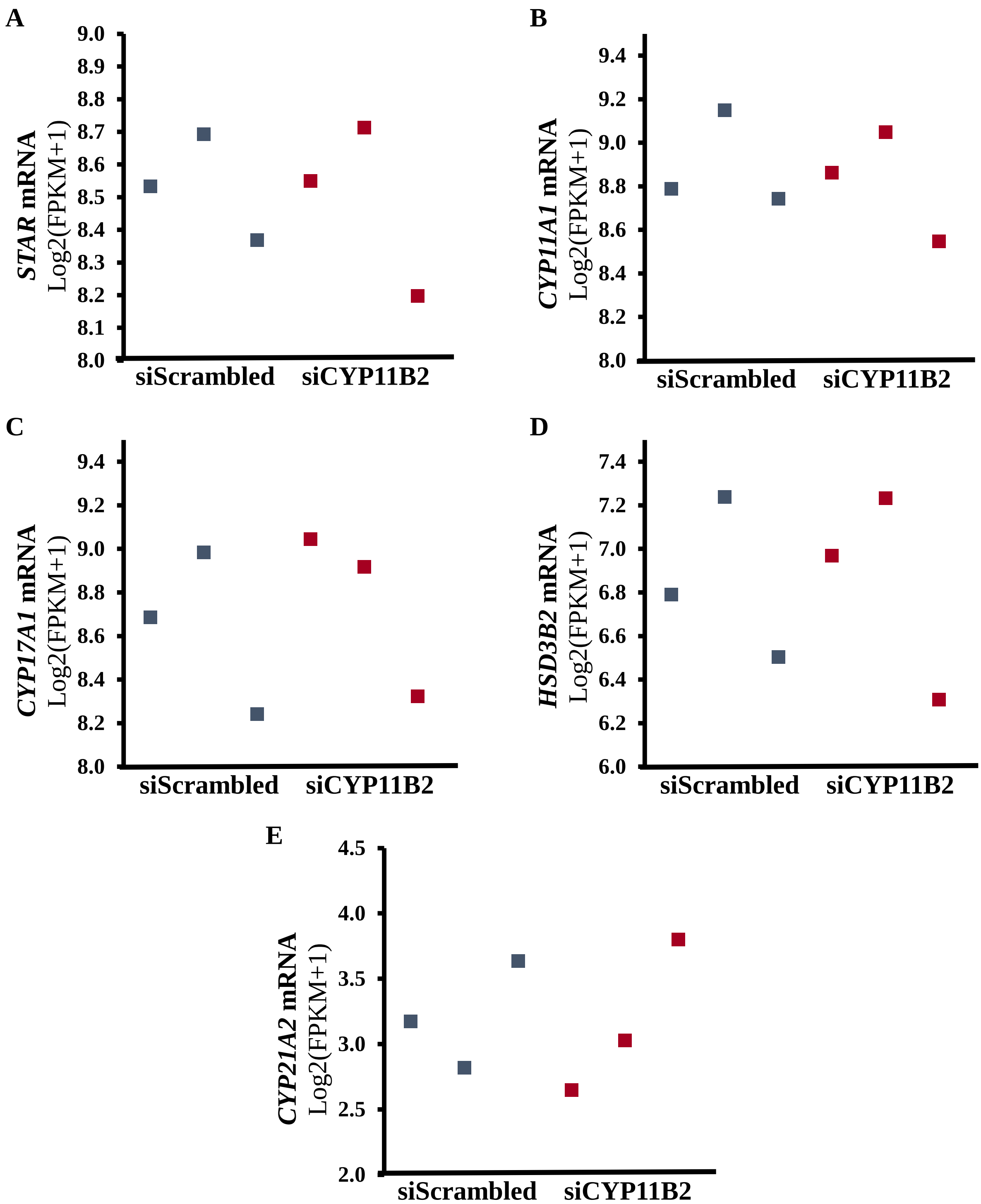

Figure S3. RNA-seq analysis of key steroidogenic genes.

STAR (A), *CYP11A1* (B), *CYP17A1* (C), *HSD3B2* (D) and *CYP21A2* (E) mRNA expression in siCYP11B2- and siScrambled-transfected HAC15 cells. None demonstrated significant differential expressions between siCYP11B2- and siScrambled-transfected cells. Data presented as log 2 of fragments per kilobase of transcript per million fragments mapped (FPKM) plus 1.

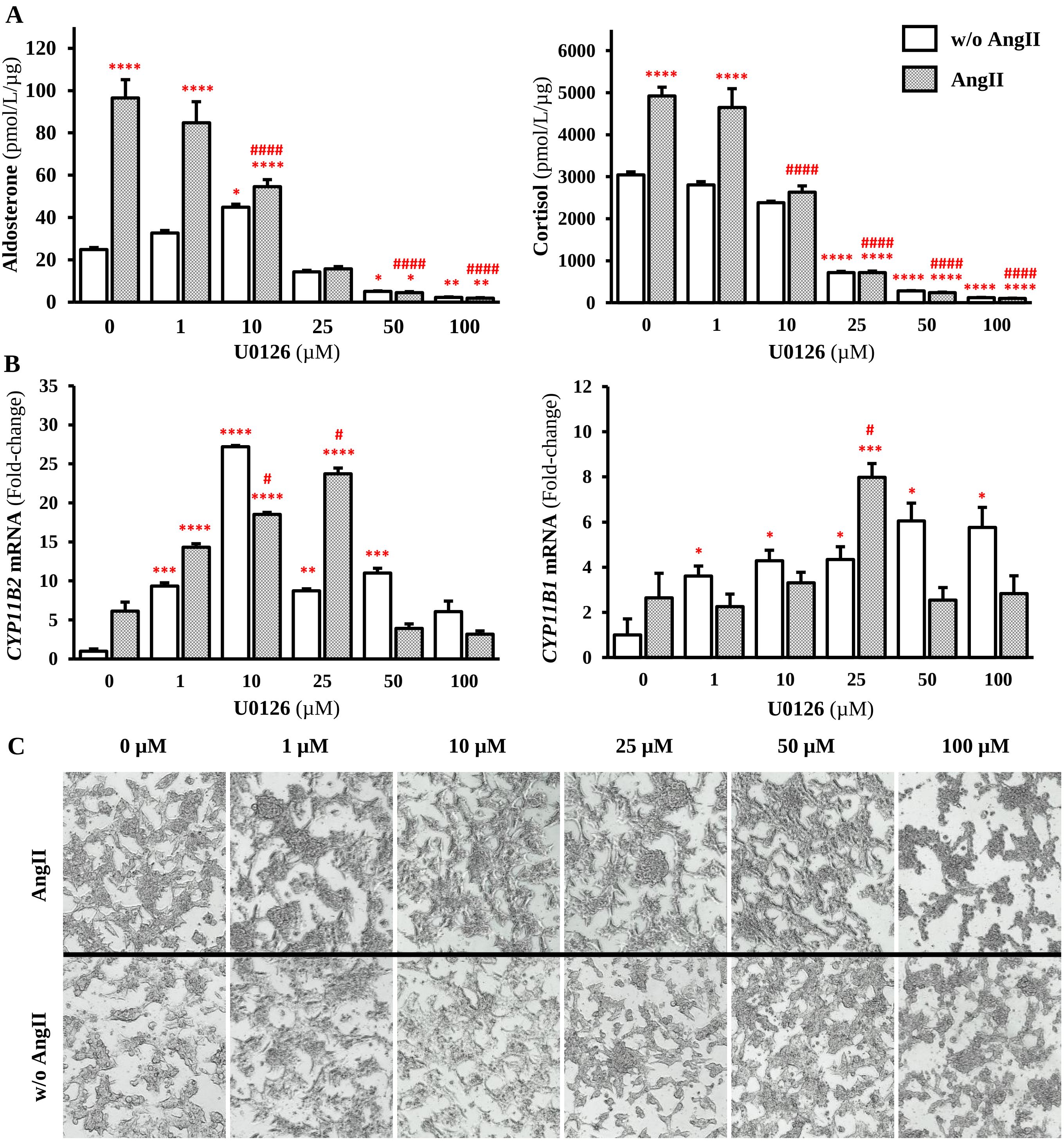

Figure S4. U0126 dose response (0 µM, 1 µM, 10 µM, 25 µM, 50 µM or 100 µM) in HAC15 cells with or without (w/o) AngII treatment.

(A) Aldosterone and cortisol production of HAC15 cell treated with 1 µM, 10 µM, 25 µM, 50 µM or 100 µM of U0126 with or with out AngII treatment measured in the supernatant collected from 24 well plate for 24 hours post-treatment in a single experiment (*n*=8). Statistical significance was calculated using 1-way ANOVA followed by Tukey post hoc test.

(B) *CYP11B2* and *CYP11B1* mRNA expression of paired cell extracts (*n*=6). Data presented as geomean of fold-change ($2^{-\Delta\Delta CT}$)±SEM. Statistical significance was calculated using Kruskal-Wallis followed by Dunn's test.

(C) 10x magnification of cells 24 hours post U0126 treatment with or without AngII treatment.

w/o, without.
*, *P*<0.05; **, *P*<0.01; ***, *P*<0.005; ****, *P*<0.001; compared to Untreated w/o AngII group.
[#], *P*< 0.05; [####], *P*<0.001; compared to Untreated with AngII group.

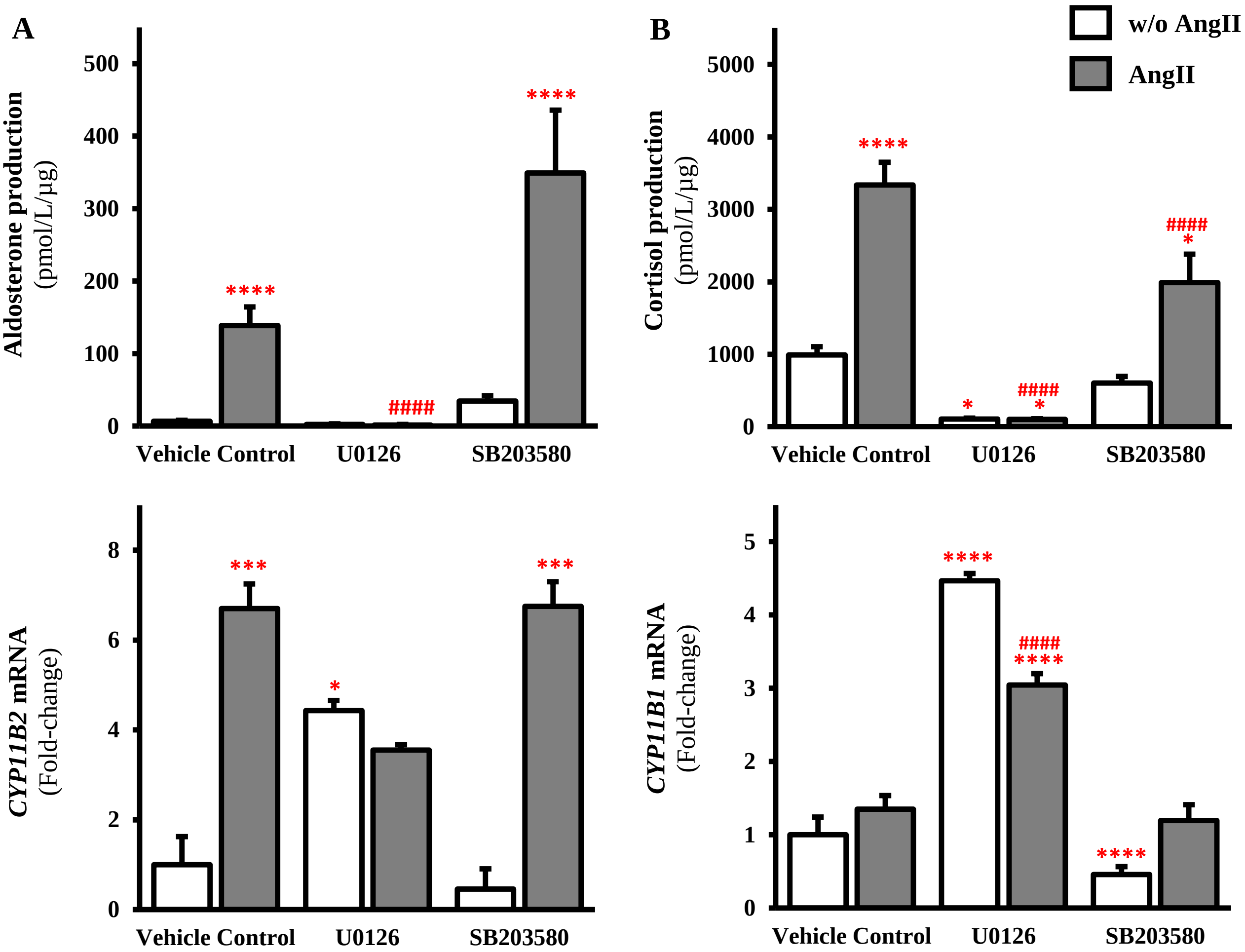

Figure S5. Effect of 50 μM U0126 or 1 μM SB203580 in HAC15 cells with or without (w/o) 1 μM AngII treatment.

(A) 24 hours aldosterone production of treated cells in supernatant from 24 well plate (*n*=11-12) and *CYP11B2* mRNA expression from the cell extracts (*n*=9). Data presented as geomean of fold-change $(2^{-\Delta\Delta CT})$±SEM.. Statistical significance was calculated using Kruskal-Wallis followed by Dunn's test for aldosterone production, 1-way ANOVA followed by Tukey post hoc test for *CYP11B2* mRNA expression.

(B) 24 hours cortisol production of treated cells in supernatant from 24 well plate (*n*=11-12) and *CYP11B1* mRNA expression from the cell extracts (*n*=9). Data presented as geomean of fold-change $(2^{-\Delta\Delta CT})$±SEM.. Statistical significance was calculated using 1-way ANOVA followed by Tukey post hoc test.

w/o, without.
*, P<0.05; ***, P<0.005; ****, P<0.001; compared to Vehicle Control w/o AngII group.
####, P<0.001; compared to Vehicle Control with AngII group.

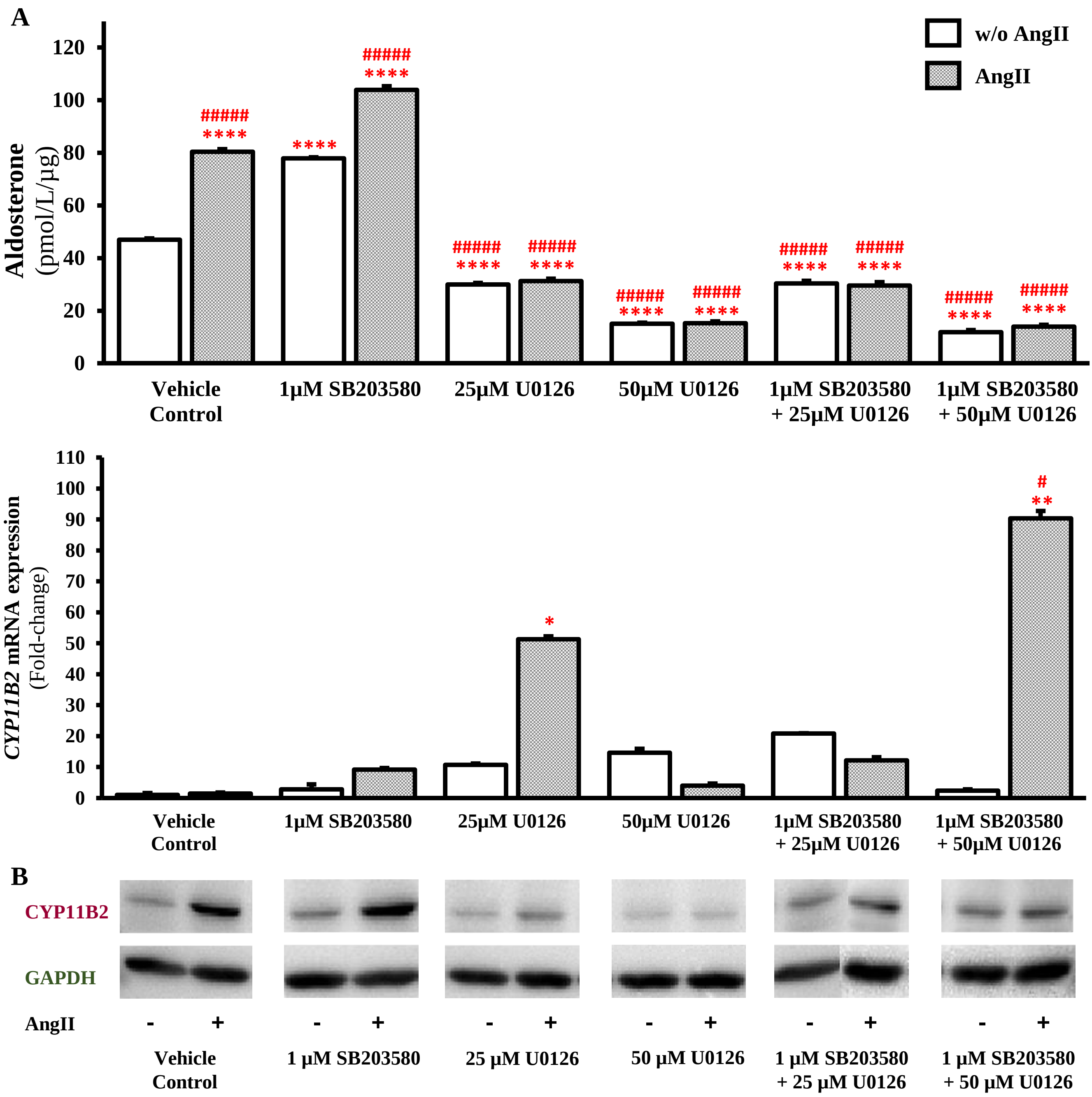

Figure S6. Co-treatment of SB203580 with U0126 in HAC15 cells in the absence or presence of AngII-stimulation.

(A) Aldosterone production in supernatants collected from HAC15 cells cultured in 24-well plates after 24 hours of treatment with 1 μM SB203580, 25 μM U0126, 50 μM U0126 or co-treatment of 1 μM SB203580 with either 25 μM U0126 or 50 μM U0126, in the absence or presence of AngII-stimulation (*n*=6), and CYP11B2 mRNA expression in corresponding cell extracts (*n*=4). Data are presented as geomean fold-change ($2^{-\Delta\Delta CT}$)±SEM. Statistical significance was assessed using one-way ANOVA followed by Tukey's post hoc test.

(B) Representative immunoblots showing CYP11B2 (upper panel) and GAPDH (lower panel) protein expression in HAC15 cells treated in (A).

w/o, without.
*, $P<0.05$; **, $P<0.01$; ****, $P<0.001$; compared to Vehicle Control w/o AngII group.
#, $P<0.05$; ####, $P<0.001$; compared to Vehicle Control with AngII group.

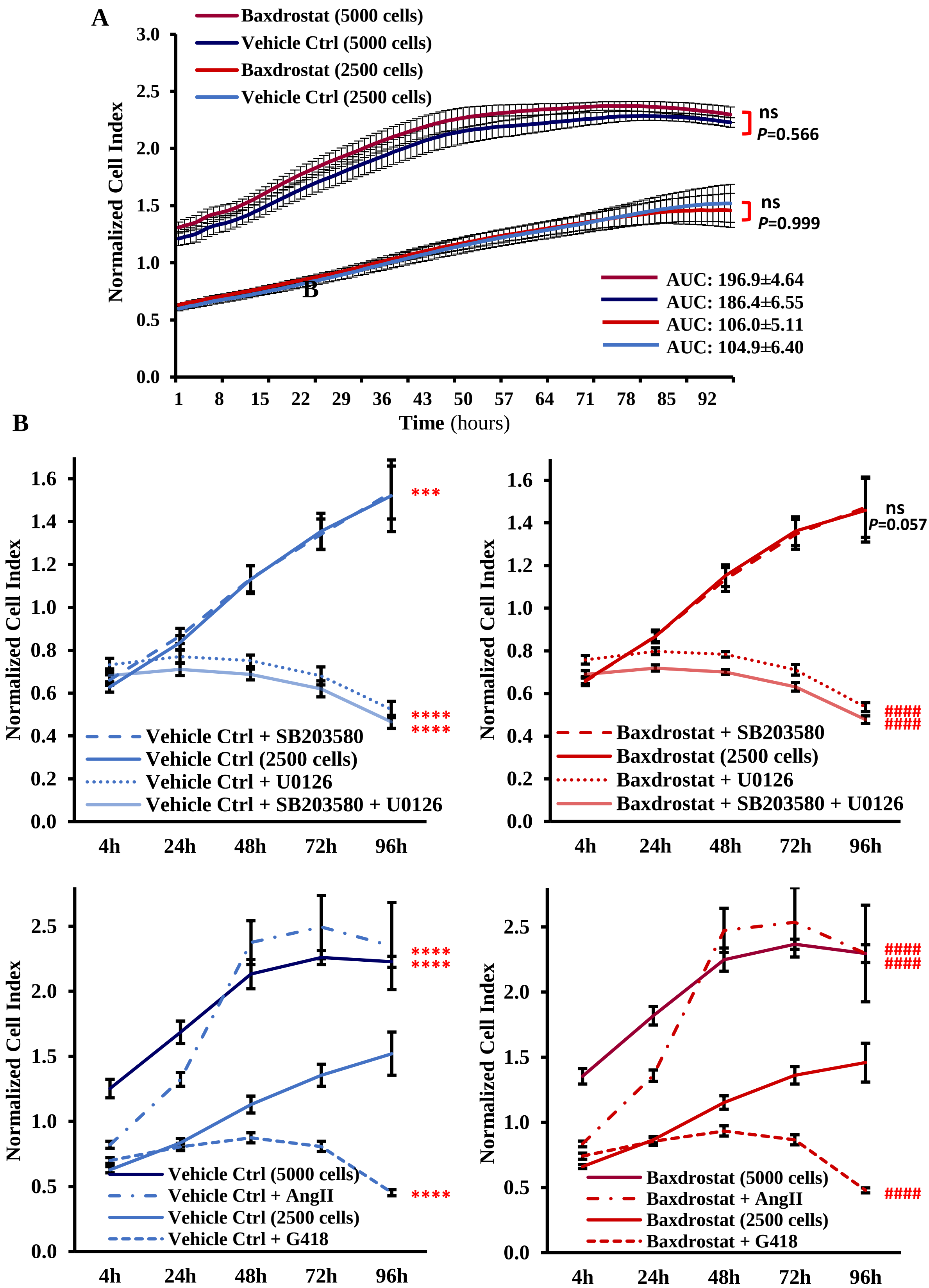

Figure S7. Effect of U0126 and SB203580 on Baxdrostat treated HAC15 cells on cell index measured by the xCELLigence analyzer .

(A) Cell index measurements for Baxdrostat or DMSO 1:1000 (Vehicle Control) (*n*=11-12) treated HAC15 cells (2500 or 5000 cells) 48 hours post-seeding, measured for 96 hours at an hour interval.

(B) Cell index measurements for Baxdrostat or DMSO 1:1000 (Vehicle Control) with treatment of 1 µM of SB203580 or 25 µM of U0126 or 400 µg/ml of G418 or 1 µM of AngII in HAC15 cells 48 hours post-seeding, measured at 4 hours, 24 hours, 48 hours, 72 hours and 96 hours timepoint (*n*=11-12). Cells were seeded at 2500 cells unless otherwise indicated (i.e. 5000 cells).

Vehicle Ctrl, Vehicle Control.
ns, not significant; compared to Vehicle Control (2500 or 5000 cells) group or Baxdrostat (2500 cells) group.
, *P*<0.005; *, *P*<0.001; compared to Vehicle Control cells (2500 or 5000 cells) group.
####, *P*<0.001; compared to Baxdrostat (2500 or 5000 cells) group.
Statistical significance was calculated using a two-way repeated measures ANOVA test.

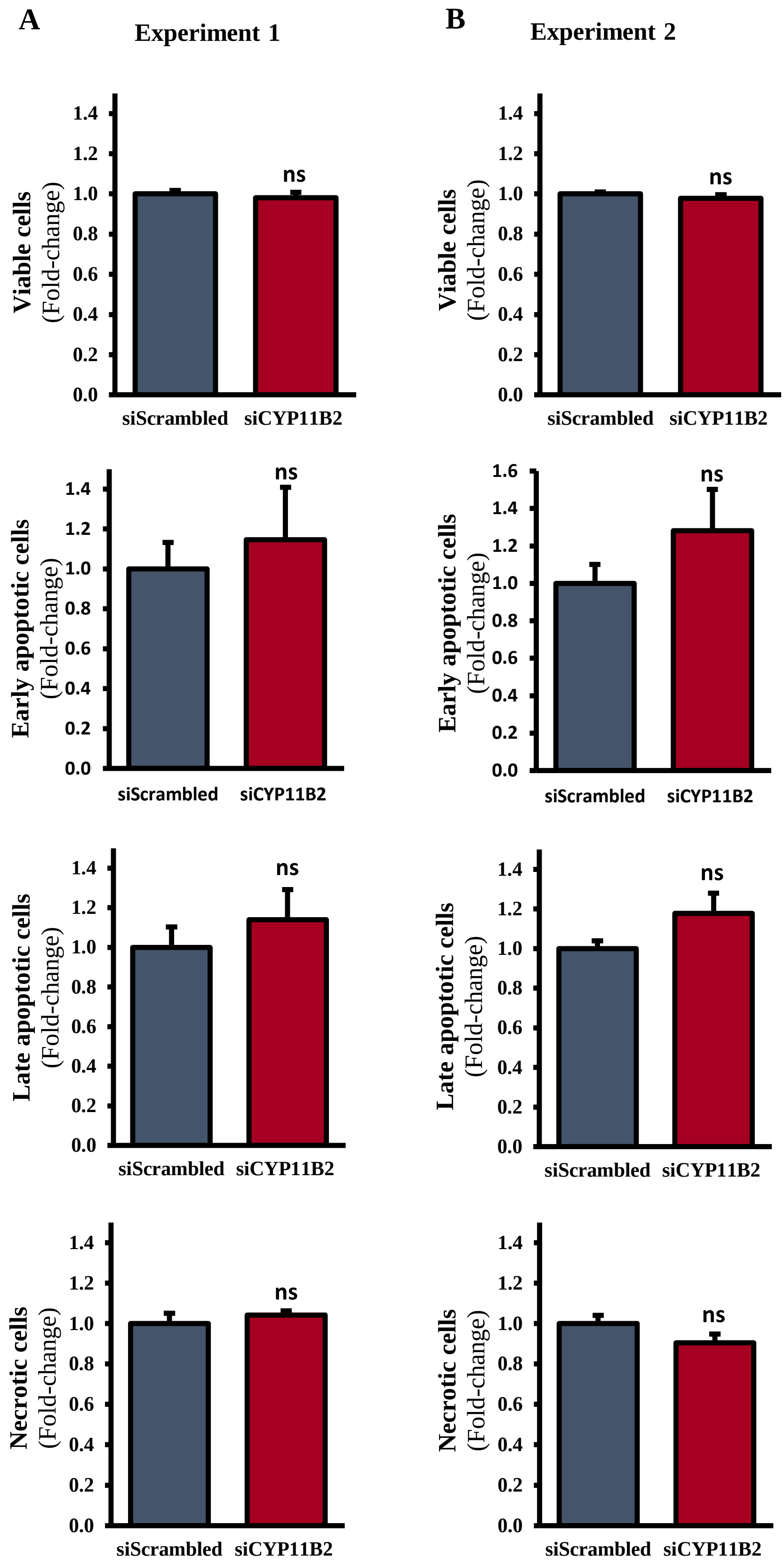

Figure S8. Flow cytometric analysis of cell viability and apoptosis in HAC15 cells 48 hours after transfection with siCYP11B2 or siScrambled.

The proportions of viable, early apoptotic, late apoptotic, and necrotic cells were measured in two independent experiments (A-B, *n*=6).

ns, not significant; compared to siScrambled group.
Statistical significance was calculated using Mann-Whitney *U* test.

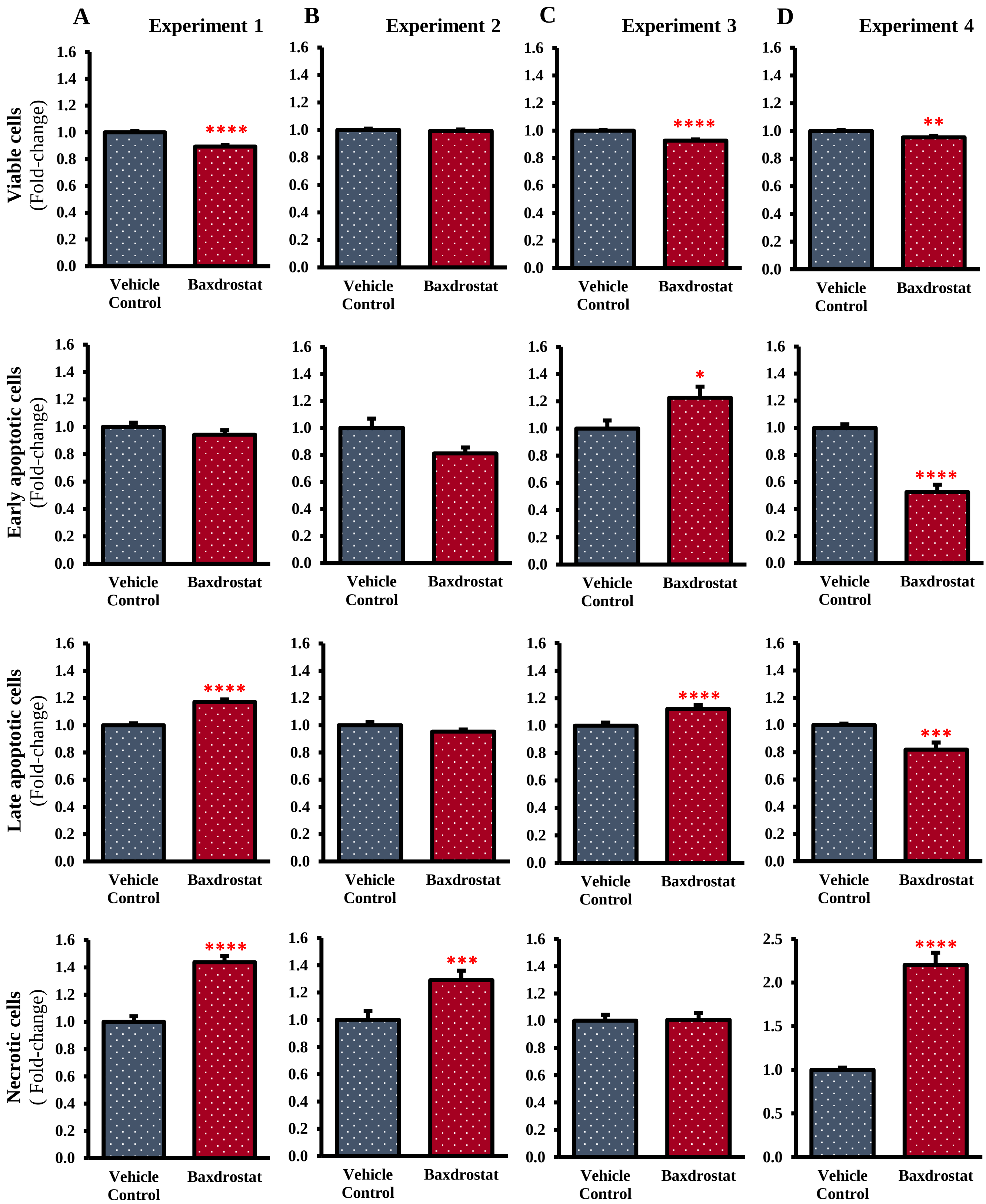

Figure S9. Flow cytometric analysis of cell viability and apoptosis in HAC15 cells 24 h after treatment with baxdrostat or vehicle control (DMSO, 1:1000).

The proportions of viable, early apoptotic, late apoptotic, and necrotic cells were measured across four independent experiments (A-D, *n*=36).

*, *P*<0.05; **, *P*<0.01; ***, *P*<0.005; ****, *P*<0.001; compared to Vehicle Control group. Statistical significance was calculated using Mann-Whitney *U* test.

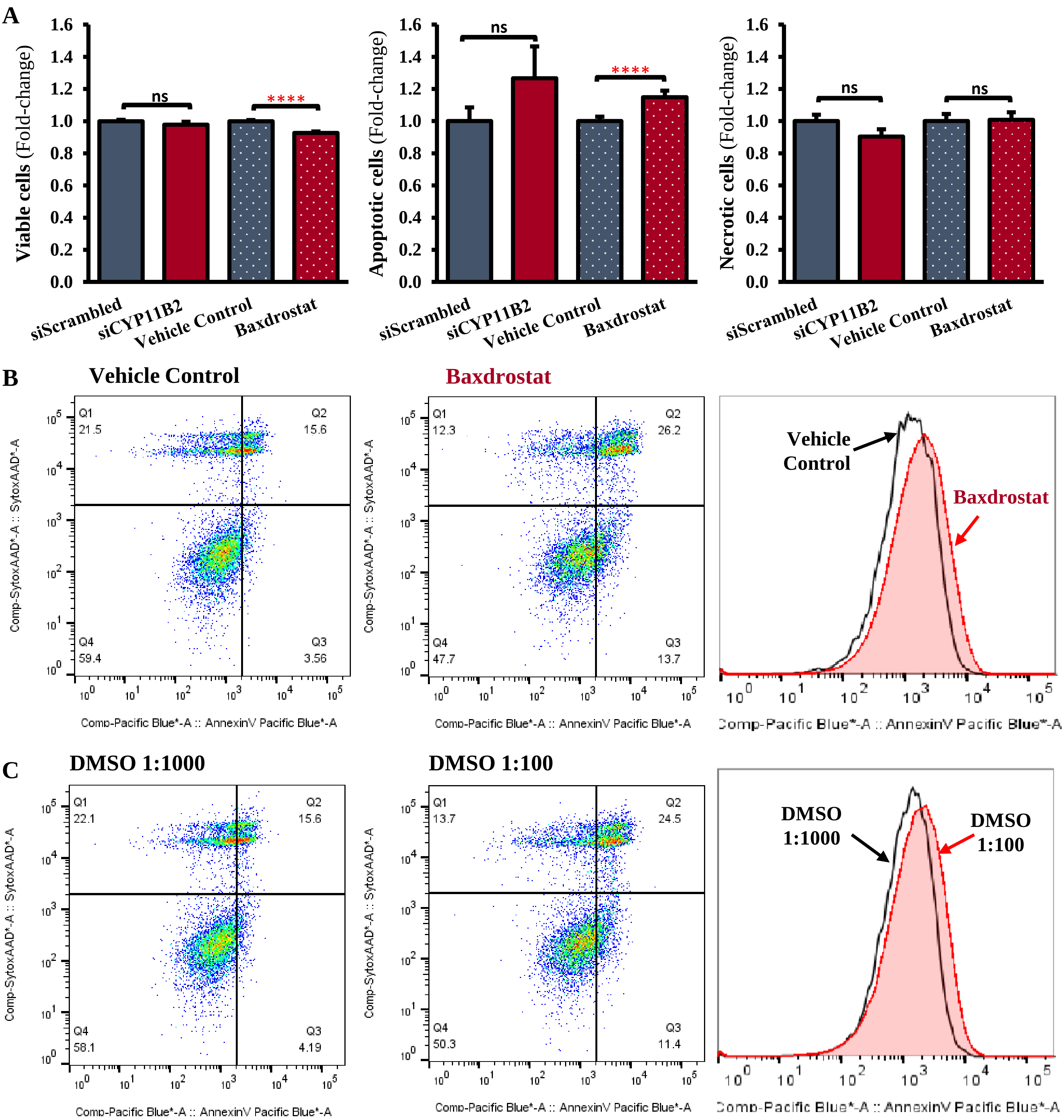

Figure S10. Baxdrostat increased apoptotic cells when no increase in necrotic cells were observed.

(A) Flow cytometry measurements fold-change of viable cells, apoptotic cells and necrotic cells in HAC15 cells 48 hours post-transfection with siCYP11B2 or siScrambled (*n*=6) and HAC15 cells 24 hours post-treatment with Baxdrostat or Vehicle Control (DMSO 1:1000) (*n*=36). Statistical significance was calculated using Student *t* test for siRNA group and Mann-Whitney *U* test for treatment group.

(B) Flow cytometry gating dot plot for Vehicle Control and Baxdrostat, and Pacific Blue AnnexinV quantification for Vehicle Control (*n*=1, black) *vs.* Baxdrostat (*n*=36, red).

(C) Flow cytometry gating dot plot for DMSO 1:1000 and DMSO 1:100 (control treatment), and Pacific Blue AnnexinV quantification for DMSO 1:1000 (*n*=1, black) *vs.* DMSO 1:100 (*n*=12, red).

ns, not significant; compared to siScrambled group or Vehicle Control group.
****, *P*<0.001; compared to siScrambled group or Vehicle Control group.

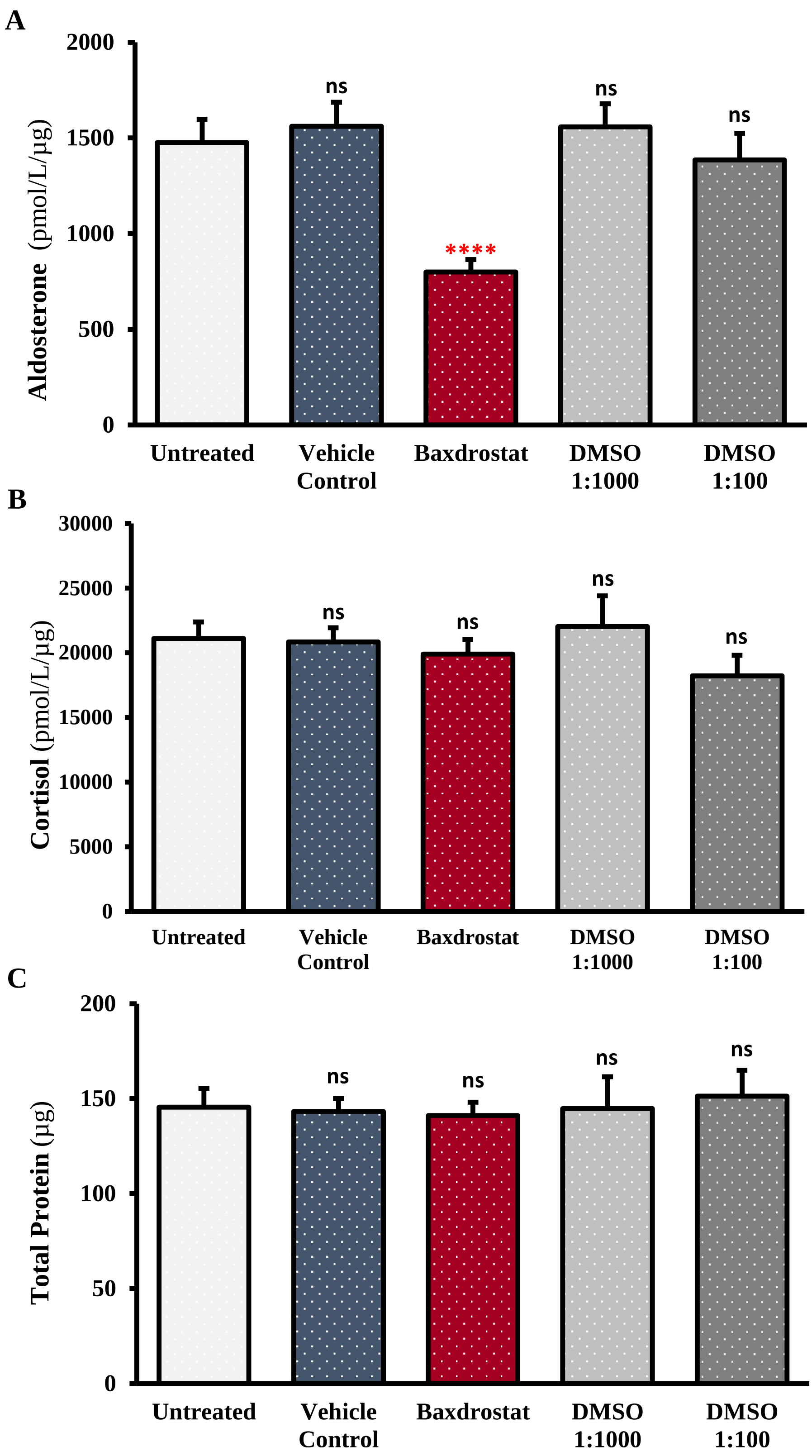

Figure S11. Baxdrostat 10 ng/untreated cells reduced aldosterone production by 45.8%±4.4.

(A) 24 hours aldosterone production of treated HAC15 cells in supernatant from 6 well plates (*n*=8-24).

(B) 24 hours cortisol production of treated HAC15 cells in supernatant from 6 well plates (*n*=8-24).

(C) Total protein of treated HAC15 cell extracts from 6 well plates 24 hours post-treatment (*n*=8-24).

ns, not significant; compared to Untreated group.
****, *P*<0.001; compared to Untreated group.
Statistical significance was calculated using 1-way ANOVA followed by Tukey post hoc test.
